# LOOP IIId of the HCV IRES is essential for the structural rearrangement of the 40S-HCV IRES complex

**DOI:** 10.1093/nar/gkv1325

**Published:** 2015-11-30

**Authors:** Jenniffer Angulo, Nathalie Ulryck, Jules Deforges, Nathalie Chamond, Marcelo Lopez-Lastra, Benoît Masquida, Bruno Sargueil

**Affiliations:** 1 CNRS UMR 8015, Laboratoire de cristallographie et RMN Biologiques, Université Paris Descartes, 4 avenue de l'Observatoire, 75270 Paris Cedex 06, France; 2 Laboratorio de Virología Molecular, Instituto Milenio de Inmunología e Inmunoterapia, Centro de Investigaciones Médicas, Escuela de Medicina, Pontificia Universidad Católica de Chile, Marcoleta 391, Santiago, Chile; 3 UMR 7156 Génétique Moléculaire Génomique Microbiologie, CNRS - Université de Strasbourg, Strasbourg, France

## Abstract

As obligatory intracellular parasites, viruses rely on cellular machines to complete their life cycle, and most importantly they recruit the host ribosomes to translate their mRNA. The Hepatitis C viral mRNA initiates translation by directly binding the 40S ribosomal subunit in such a way that the initiation codon is correctly positioned in the P site of the ribosome. Such a property is likely to be central for many viruses, therefore the description of host-pathogen interaction at the molecular level is instrumental to provide new therapeutic targets. In this study, we monitored the 40S ribosomal subunit and the viral RNA structural rearrangement induced upon the formation of the binary complex. We further took advantage of an IRES viral mutant mRNA deficient for translation to identify the interactions necessary to promote translation. Using a combination of structure probing in solution and molecular modeling we establish a whole atom model which appears to be very similar to the one obtained recently by cryoEM. Our model brings new information on the complex, and most importantly reveals some structural rearrangement within the ribosome. This study suggests that the formation of a ‘kissing complex’ between the viral RNA and the 18S ribosomal RNA locks the 40S ribosomal subunit in a conformation proficient for translation.

## INTRODUCTION

**Figure 1.**
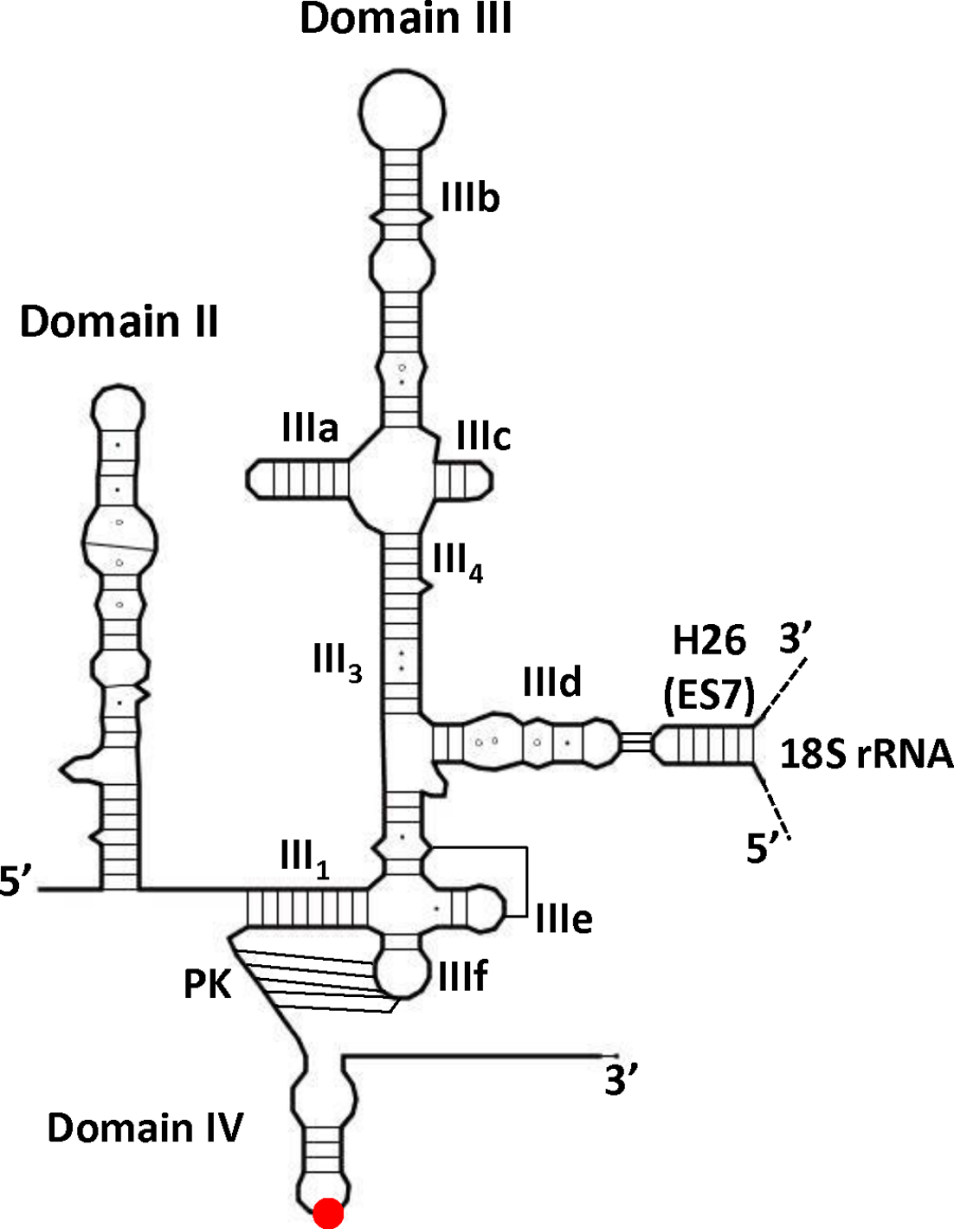
Line drawing of the secondary structure of HCV IRES domain II and III. Canonical Watson-Crick base pairs are represented by lines, G-U base pairs by black dots and non-canonical interactions by hollow circles. The AUG initiation codon is represented by a red dot. The name of the different subdomains of domain III (IIIa-f), the helices III3 and III4, the pseudoknot (PK) and domain IV are specified. The ‘kissing-loop’ between the IIId loop and ES7-h26 of the 18s rRNA is schematized.

Understanding the host-pathogen interactions is a critical issue for the development of preventive and curative therapies against viruses. Of particular interest is the identification of the molecular structures and mechanisms allowing these obligatory intracellular parasites to subvert cellular machines to ensure their replication. In addition, such phenomena provide a unique opportunity to explore physiological and cellular processes at the molecular level. Notably, many viruses have been shown to manipulate translation initiation to further their replication. These viral systems provide simplified paradigms that can be used to untangle the functional and structural aspects of this complex process. Translation initiation results in the positioning of a translation-competent 80S ribosome on the *bona fide* initiation codon. For eukaryotes, a minimal set of 10–14 cellular protein factors, known as eukaryotic Initiation Factors (eIFs), are required to ensure the 40S subunit recruitment at the 5′ cap terminus of the mRNA, its migration to the initiation codon, and the 80S ribosome assembly (1,2). Translation initiation of some viruses proceeds through a ‘*simplified*’ mechanism known as internal entry of the ribosome during which the initiation complex is directly recruited on or at the vicinity of the translation start codon (1,3–6). Several different ribosome internal entry mechanisms have been described, with all of them relying on a sequence upstream of the initiation codon (with one exception (7–9)). This sequence is known as an IRES, for Internal Ribosome Entry Site. In the best characterized cases, the three dimensional structure of the viral mRNA appears to be crucial, by functionally replacing some of the eIFs (10–15). Not surprisingly, the direct interaction of the IRES with the small subunit of the ribosome could be a common feature of all IRESes (6). This is for instance the case of the Human Hepatitis C Virus (HCV) uncapped viral mRNA on which translation initiation occurs with a minimal set of initiation factors. In a first step, the HCV-IRES forms a stable complex with the 40S ribosomal subunit which then binds the eIF2 ternary complex (Met-tRNA_i_^Met^-GTP-eIF2) to yield a preinitiation complex that is paused on the initiation codon. The transition to an 80S translation competent ribosome is then ensured by eIF5, eIF5B and the 60S ribosomal subunit. Alternatively, under conditions where eIF2 is inactivated, the HCV-IRES is able to promote eIF2- and eIF5-independent translation initiation. under such conditions, several distinct mechanisms ensuring tRNA_i_^Met^ delivery to the initiation codon have been reported, involving either eIF5B and eIF3 (16,17) or eIF2D/ligatin factor (18,19), or even eIF2A (20). The IRES also binds eIF3 with a high specific activity, but a recent structural and functional study on an HCV-like IRES strongly suggests that this interaction is not involved in the initiation mechanism *per se*(11). IRES binding to the small ribosomal subunit would rather pull eIF3 away from the 43S complex, thus unmasking the 40S IRES binding site. In addition, it has been suggested that such interaction would titrate eIF3, further reducing the canonical cellular translation (11). The IRES function relies on a 330 nucleotide long sequence within the 5′ Untranslated Region (5′ UTR) which adopts a secondary and tertiary structure that has been extensively studied. The IRES has been subdivided into three domains: domain II is mostly involved in the step leading from the 48S-like preinitiation complex to the 80S complex; domain III directly recruits eIF3 and the small ribosomal subunit, and domain IV harbors the initiation codon. Domain III is divided in several helices numbered IIIa to IIIf and contains a pseudoknot (PK) (Figure 1). The IRES holds a tRNA like domain as monitored by RNase P cleavage, although it has not been shown to be a determinant of ribosome binding (21,22). Most interestingly cryo-EM, Single Molecule FRET and functional studies have shown that the HCV-IRES not only binds the small ribosomal subunit but that a structural rearrangement of the 40S/HCV-IRES complex is required for translation to occur (23–28).

Early studies suggested that the domain III is crucial for translation (29–33) and more precisely that stem-loop IIId is required for the formation of the 40S/IRES complex (30,31,34–36). Indeed, three phylogenetically conserved guanine residues within loop IIId (37) have been shown to base pair with the apical loop of the extension segment 7 within the 18S rRNA h26 helix (ES7^S^)(11,38–40). Here, we focused on the study of IIId mutant IRESes originally identified in patient isolates that exhibits a defect in translation (29). We further examined the translation efficiency of this mutant in diverse contexts and characterize the initiation complexes formed. We used selective 2′-hydroxyl acylation analyzed by primer extension ‘SHAPE’ RNA probing technology to define the impact of mutations on the IRES structure and on the coordinated structural rearrangement of both the IRES and the 18S ribosomal RNA upon binary complex formation. A three-dimensional whole atom model was also built in order to use nucleic acid stereochemistry as a supplementary constraint to better characterize the key features of the IRES binding to the 40S subunit. While this work brings some new information on the IRES structure and its rearrangement upon ribosome binding, it further shows that translational deficient loop IIId mutants are still able to bind the 40S ribosomal subunit, but cannot appropriately manipulate the ribosome to promote translation. Altogether our results strongly suggest that the loop IIId-ES7^S^ interaction triggers a structural rearrangement of the 40S/HCV-IRES complex that is required for HCV mRNA translation.

## MATERIALS AND METHODS

### *In vitro* transcription

Uncapped monocistronic RNAs were directly transcribed using T7 RNA polymerase from PCR products containing the T7 RNA polymerase promoter (41). For this, the wild type and mutant HCV IRES, fused with the coding region of firefly luciferase (FLuc), were amplified from the dl HCV 1b, dl G266A, dl G_268_U or dl G_266_A/G_268_U plasmids (29), using the primers FV30 5′ CCATATGTAATACGACTCACTATAGGGTTGGGGGCGACACTCC 3′ (from nucleotide 1672 of the plasmid) and ClaI-FlucR 5′- ATCGATTACACGGCGATCTTTCCG -3′ (until nucleotide 3701 of the same plasmid). The RNAs were synthesized by *in vitro* transcription conducted in a final volume of 100 μl, using T7 RNA polymerase, 5 mM DTT, 5 mM rNTPs, 1X transcription buffer (40 mM Tris–HCl pH 8.0, 25 mM MgCl_2_, 1 mM spermidine) and 20 U of RNAsin (Promega) and incubated 2 h at 37°C. Upon synthesis, RNAs were treated with 2 U of DNAse RQ1 (Promega) for 30 min at 37°C. RNA was precipitated for one hour with 2.5 M LiCl, centrifuged at 16 000 *g* for 30 min at 4°C, washed with 70% ethanol, resuspended in 50 μl of nuclease-free water and purified through Sephadex G50 columns. RNAs concentrations were determined spectrophotometrically (Biospec-NanoDrop technology), and RNA integrity was monitored by electrophoresis on agarose gels. Radiolabeled uncapped monocistronic RNAs were transcribed as above but in presence of 1 mM of UTP and 3000 mCi/mmol of α-^32^P-UTP.

### *In vitro* translation

Unless indicated 1 pmol of an *in vitro* transcribed RNAs, were translated in 60% (v/v) nuclease-treated Flexi®-rabbit reticulocyte lysate (Flexi^®^-RRL; Promega), supplemented with 0.5 mM magnesium acetate, 100 mM potassium chloride and 0.02 mM amino acids in 10 μl. The reaction was incubated for 60 min at 30°C. Firefly luciferase activities were measured in a single tube using the Luciferase Assay System (Promega) on a Sirius luminometer (Berthold Detection Systems) or an Infinite 200 pro microplate reader (Tecan), according to the manufacturers’ protocol.

For ^35^S-methionine labeling, monocistronic RNA (1 pmol) was translated as above, but in the presence of 0.02 mM of amino acid lacking methionine and supplemented with 0.6 mCi/ml [^35^S]-methionine. Translation reactions were incubated for 60 min at 30°C. The reaction was stopped with 90 μl of protein loading buffer (10% SDS, 50 mM Tris pH 6.8, 10% glycerol, 100 mM DTT and 0.1% bromophenol blue). Ten microliters of the final mix was loaded and resolved by SDS-PAGE (12%). The gel was fixed for 20 min in a solution of 30% of ethanol and 10% of acetic acid, and dried for 60 min in a vacuum drying system at 75°C. The labeled products were visualized and quantified using a Storm PhosphorImager (GE Healthcare).

*In vitro* translation in cellular extracts, were performed in a 10 μl reaction using 50% (v/v) of HuH7 translational extracts (42), programmed with 0.5 pmol of monocistronic RNA, 10% of master mix (10 mM ATP, 2 mM GTP, 100 mM creatine phosphate, 1 mg/ml creatine phosphokinase, 0.2 mM amino acids, 125 mM HEPES–KOH, pH 7.3), 0.25 mM spermidine, 4 U of RNase Inhibitor (Thermo Fisher Scientific), 75 mM of potassium acetate, 1 mM magnesium chloride. The translation was conducted for 90 min at 30°C. FLuc activities were measured as above.

### 3D modeling

The whole atom models were generated using Assemble (43). In brief, the cryo EM structure of the CSFV IRES bound to the 40S ribosomal subunit and eIF3 (11) was used as a template to model the HCV IRES. The *Tetrahymena thermophila* (T.th) 40S subunit (44) was then docked into the cryo EM map of the rabbit 40S bound to the CSFV IRES. The T.th 18S rRNA was humanized by replacing the natural h26 sequence by the human one, encompassing nucleotides 1101–1131 (human numbering). It is worth noting that domain IV was unwound while the pseudoknot remained closed as in (26). The SHAPE protection pattern was fulfilled by undertaking very minor adjustments between the models of the IRES and of h26. Our model was built blind with respect to the 40S-bound HCV IRES cryo EM structure from the Ban group (40) in order to characterize the structural features that could be deduced from existing biochemical data. Superimpositions were carried out in Chimera (45). Pictures were designed using Pymol (46).

### Quantification of RNA by qRT-PCR

The HCV-FLuc mRNA from translation reactions was detected by a one-step reverse transcription (RT)-polymerase-chain reaction (PCR), using the SuperScript™ III one-step RT-PCR system with Platinum^®^ Taq DNA Polymerase (Life Technologies), following the manufacturer's instructions. The amplification (nucleotides 167–302 of the Fluc coding region) was achieved using the sense primer: FLucS-qPCR 5′ ACTTCGAAATGTCCGTTCGG 3′ and the anti-sense primer: FlucAS-qPCR 5′ GCAACTCCGATAAATAACGCG 3′. *In vitro* transcribed RNA (T7 RNA polymerase; Fermentas) generated from plasmid dl HCV 1b (29), was used as a positive control, while water, and an unrelated RNA, were used as negative controls.

### Preparation of 40S ribosomal subunits and initiation factors

Ribosomal subunits were prepared following previously established procedures (47,48). Cytoplasmic extracts from HeLa cells (Ipracell) or rabbit reticulocyte lysate (Green Hectares) were centrifuged in Buffer A (20 mM Hepes-KOH pH 7.5, 50 mM KCl, 4 mM MgCl_2_, 2 mM DTT and 0.25 M sucrose) at 14 000 × *g* for 15 min to remove mitochondria. The supernatant was layered onto a 50% sucrose cushion and centrifuged for 5 h at 44 000 rpm in a 45 Ti rotor. The pellet (P1) was further resuspended in Buffer A (OD_260_ ± 100) and treated with 1 mM puromycin (Sigma) for 10 min on ice followed by 10 min at 37°C. KCl was then added to a final concentration of 500 mM and the solution was incubated on ice for 30 min. The suspension was centrifuged for 1 h 40 at 70 000 rpm in a 70 Ti rotor. The obtained pellet (P2) was resuspended in Buffer B (20 mM HEPES–KOH pH 7.5, 0.5 M KCl, 4 mM MgCl_2_ and 2 mM DTT) and layered onto a 10–30% sucrose gradient prepared in Buffer B. Gradients were run for 17 h at 22 000 rpm in a SW32 Ti rotor. Fractions were collected and 10 μl of each was loaded onto an agarose gel. Fractions corresponding to 40S ribosomal subunits were pooled, dialyzed and concentrated in Buffer C (20 mM HEPES KOH pH 7.5, 100 mM KCl, 2 mM MgCl_2_, 2 mM DTT and 0.25 M sucrose). eIF3 was prepared from HeLa cells extract as previously described (49). eIF2 was purified from rabbit reticulocyte lysate as previously described (47). The identity of both proteins was checked by mass spectrometry analysis, eIF3 was tested for its ability to specifically bind eIF3, and both proteins were tested for their ability to promote initiation complex formation on EMCV IRES. tRNA_Met_^i^ was transcribed and charged *in vitro* as described (47). The charging efficiency was monitored on a 20% denaturing page using labeled ^35^S-methionine.

### Mobility of complexes during sucrose density gradients centrifugation

Complexes were assembled on ^32^P-labeled HCV IRES-FLuc mRNA or IRES RNA wild type and mutants. 2 pmol of RNA (10 μl) was denaturated by heating the RNA to 85°C for 2 min, followed by cooling to room temperature for 7 min. Determination of complex formation with purified 40S ribosomal subunits (HeLa or RRL) was performed by incubating the RNA (20 nM) with 400 nM of 40S subunits for 20 min at 30°C in FB buffer (20 mM Tris pH 7.5, 100 mM KOAc, 200 mM KCl, 1 mM DTT, 2.5 mM MgCl_2_). 80S and 48S like complex analysis were carried out as in (10) using flexi-RRL. All the reactions were stopped on ice and layered over 15–50% or 10–30% sucrose gradients (25 mM Tris pH 7.6, 6 mM MgCl_2_, 75 mM KCl) and sedimented by ultracentrifugation at 39 000 rpm for 4 h at 4°C in a SW40 Ti rotor (Beckman Coulter). Fractions of 300 μl were collected. 100 μl of each fraction were vacuumed blotted onto a Hybond N+ membrane (Amersham), exposed overnight and quantified using a Storm PhosphorImager (GE Healthcare). The amount of RNA in each fraction was determined and expressed as the percentage of total counts.

### Filter binding assay

Radiolabeled RNA (10 nM) was denatured by heating to 80°C for 2 min followed by cooling to room temperature in FB buffer (20 mM Tris–Cl pH 7.5, 100 mM KOAc, 200 mM KCl, 2.5 mM MgCl_2_, 2 mM DTT). Serial dilutions from a 2 μM solution of 40S were prepared extemporaneously, added to a 10 μl reaction and incubated at 37°C for 10 min. The reactions were then used for filter binding assays. Filter binding was accomplished essentially as previously described using two filters (34). From top to bottom a nitrocellulose filter and a charged nylon filter. The filters were presoaked in FB buffer, assembled in the dot blot apparatus and the reactions were applied and directly vacuum filtered. The filters were then rinsed with FB buffer, removed and radioactivity was quantified using a storm phosphorImager (GE Healthcare). To determine the apparent *K*_d_, the data was fit to the Langmuir isotherm described by the equation *θ* = *P*/[*P* + *K*_d_] where *θ* is the fraction of RNA bound and P is 40S subunit concentration.

### IRES probing and ribosome footprinting

IRES SHAPE probing with 5 mM of 1-methyl-7-nitroisatoic anhydride (1M7) was performed as previously described (50,51) in presence or absence of 300 nM of 40S ribosomal subunit (10). RNAs were reverse transcribed using the primer fluc3 (5′ GGAACCAGGGCGTATCTCTTCATAGC3′) complementary to the fluc coding region and labeled with WellRed D2-PA or D4-PA (Sigma Aldrich). For 18S probing and footprinting : 25 pmol of HCV IRES RNA (wild type or double mutant) were resuspended in water, denatured for 2 min at 80°C and cooled to room temperature for 10 min in Buffer B (20 mM HEPES pH 7.5, 100 mM KOAc, 2.5 mM of MgCl_2_). 10 pmol of purified 40S ribosomal subunits from HeLa cells were added and the mixture was incubated for 20 min at 37°C. Then, 5 mM of 1M7 or the equivalent volume of dimethyl sulfoxide (DMSO) was added, and the mixture was incubated at 37°C for 3 min. Treated RNAs were extracted as follows: to each sample, 200 ul of GTC-Phenol Mix (2.5 M Guanidinium ThioCyanate, 76 mM β-mercaptoethanol, 1% N-lauryl sarcosine, 60% phenol pH7) was added and the samples were heated at 65°C for 10 min, then placed on ice for another 10 min. 54 ul of NaOAc pH 5.2 100 mM and 100 ul of chloroform:isoamyl alcohol (24:1) were added. The samples were mixed and centrifuged at 14 000 rpm for 5 min. The upper phase was recovered and precipitated with 1 ml of 100% ethanol, washed twice with 70% ethanol and the pellet was resuspended in 10 μl of nuclease free water. Modified and unmodified 18S rRNAs were used for a reverse transcription reaction using the following primer (5′ GTCAAATTAAGCCGCAGGCT 3′) complementary to the human 18S rRNA and 5′ labeled with WellRED D2-PA or D4-PA (Sigma Aldrich). Briefly, in all SHAPE reactions RNAs were heated for 2 min at 95°C in the presence of 2 μl of DMSO and cooled on ice for 1 min. Three μl of primer (2 μM) were added to each reaction and incubated at 65°C for 5 min, then at 35°C for 10 min to allow the annealing of the primers and cooled on ice. Modifications were revealed by reverse transcription using RNase H M-MLV RT (Promega) and the cDNA fragments were resolved by capillary gel electrophoresis (Beckman Coulter CEQ 8000). Data were interpreted and analyzed using the software QuShape (52).

RNAs probing was performed at least in triplicate with distinct RNA and ribosome preparations, enabling to assess the reactivity differences using a statistical approach (*t*-test). The statistical significance was not the only criterion used to assess the structure probing relevance. We defined a set of criteria in which the reactivities of a given nucleotide in two different situations were considered to be different (i.e. considered as a footprint) if *t* < 0.05, if the mean reactivities differ by at least 1.5-fold, and if their absolute difference is at least 0.1. These positions are marked with full triangles and can detect subtle modifications revealing significant differences even though 2′ OH group reactivities fall in the same category as represented by the color code on the sequence. We also defined a second set of criteria which reveals positions for which the SHAPE value may be more variable but that are very strongly protected or exposed upon mutations or ribosome addition (*t* test < 0.15, ratio > 2.25 and difference > 0.15). Such positions marked with hollow triangles are potentially involved in tertiary or intermolecular contacts and are therefore of interest (53).

## RESULTS

### Mutations in loop IIId strongly affect translation

We previously reported the isolation and cloning of 54 naturally occurring IRES variants from HCV viruses circulating in patients’ peripheral blood (29). Among those, a genotype 5a isolate bearing three mutations, G_137_U, G_266_A and G_268_U, was found to be very inefficiently translated (∼10% of the wild type construct). The two mutations in the conserved loop IIId were introduced separately (G_266_A or G_268_U) or together (G_266_A/G_268_U), designated below as the ‘double mutant’ (DM) in the HCV genotype 1b context and were shown to be responsible for the observed phenotype (29). To further characterize the effect of these mutations, the Firefly luciferase open reading frame (ORF) was placed 45 nt downstream from the WT or mutant HCV IRES initiation codon. *In vitro* translation assays were performed showing that the chimeric ORF used does neither interfere with the relative translation efficiency of the WT HCV IRES nor with the phenotype of the mutants. Nonetheless, the constructs contain two initiation codons: the *bona-fide*_342_AUG_344_ triplet in HCV IRES domain IV (_342_AUG_HCV_) followed by the Firefly luciferase initiation codon (_390_AUG_luc_). To avoid any ambiguity in the interpretation of our data, we confirmed experimentally that the observed translation is initiated on the *bona fide*_342_AUG_HCV_ codon (Supplementary Figure S1).

**Figure 2.**
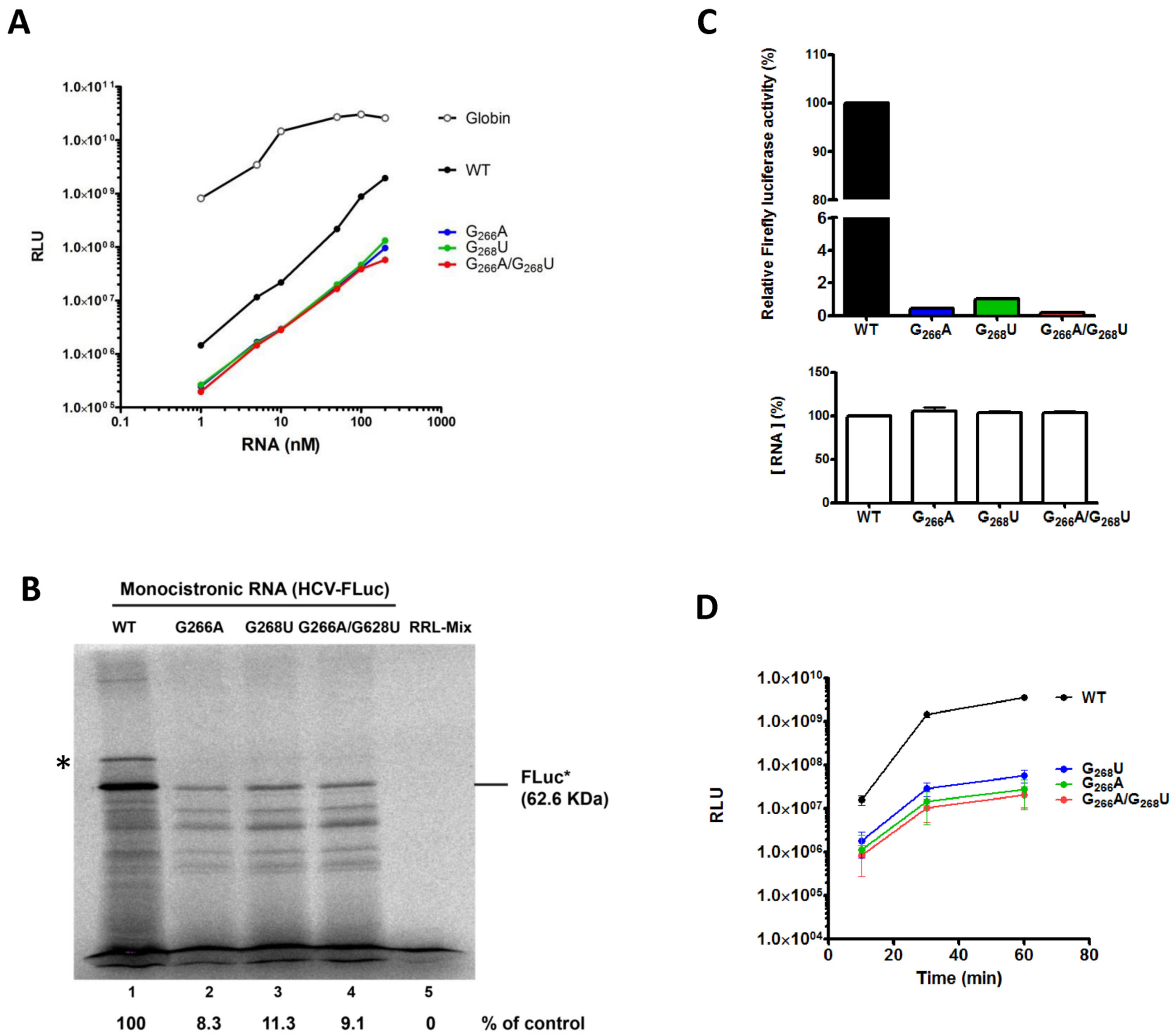
Effects of loop IIId mutants on *in vitro* translation. Different amounts (**A**) or 1 pmol (**B**) of uncapped monocistronic WT (black) or G_266_A (green) or G_268_U (blue) or DM (G_266_A/G_268_U, red) RNA were used to program Rabbit Reticulocyte Lysate (RRL). The *in vitro* translated products were measured by using the Luciferase Assay System (Promega) and expressed as Relative Luminescence Unit (**A**) or resolved by SDS-PAGE and incorporation of ^35^S-methionine was quantified. The HCV-Fluc (F-Luc*) fusion protein position is indicated. The asterisk (*) denotes a band corresponding to an artifactual migration of F-Luc* (see Supplementary Figure S1) (**B**). (**C**) 0.5 pmol of uncapped monocistronic WT (black) or G_266_A (blue) or G_268_U (green) or DM (G_266_A/G_268_U, red) RNA were used to program HuH7 translational extracts (see Materials and Methods). Firefly luciferase activity was measured and expressed relative to the WT control (upper panel). The amount of RNA translated was ascertained by qRT-PCR and expressed relative to the WT control. The results are the mean of at least three independent experiments ± standard deviation. (**D**) 0.1 μM of uncapped monocistronic WT (black) or G_266_A (blue) or G_268_U (green) or DM (G_266_A/G_268_U, red) RNA were used to program RRL. Firefly luciferase activity was measured at different time points and expressed as relative luminescence units (RLU). The results are the mean of at three independent experiments ± standard deviation.

In order to better characterize the translation rates, we first investigated the dependency between RNA concentration and translation efficiency in RRL (Figure 2). The monocistronic wild type, G_266_A, G_268_U and DM constructs were transcribed as uncapped RNAs, and increasing concentrations (1 to 200 nM) were used to program RRL. As previously observed, G_266_A, G_268_U and DM drastically reduced the translation efficiency down to 1–10% relative to WT (Figure 2A-B). We showed that this effect was independent of the translation system by using translation competent extracts generated from the hepatic cell line Huh7 (Figure 2C). To gain further insights into the origin of the mutant deficiency, we next evaluated their translation kinetics in RRL. As can be observed in Figure 2D, the translation deficiency displayed by the mutants can be observed as early as after 10 min of translation.

### Monitoring the effects of loop IIId mutations on initiation complex formation

**Figure 3.**
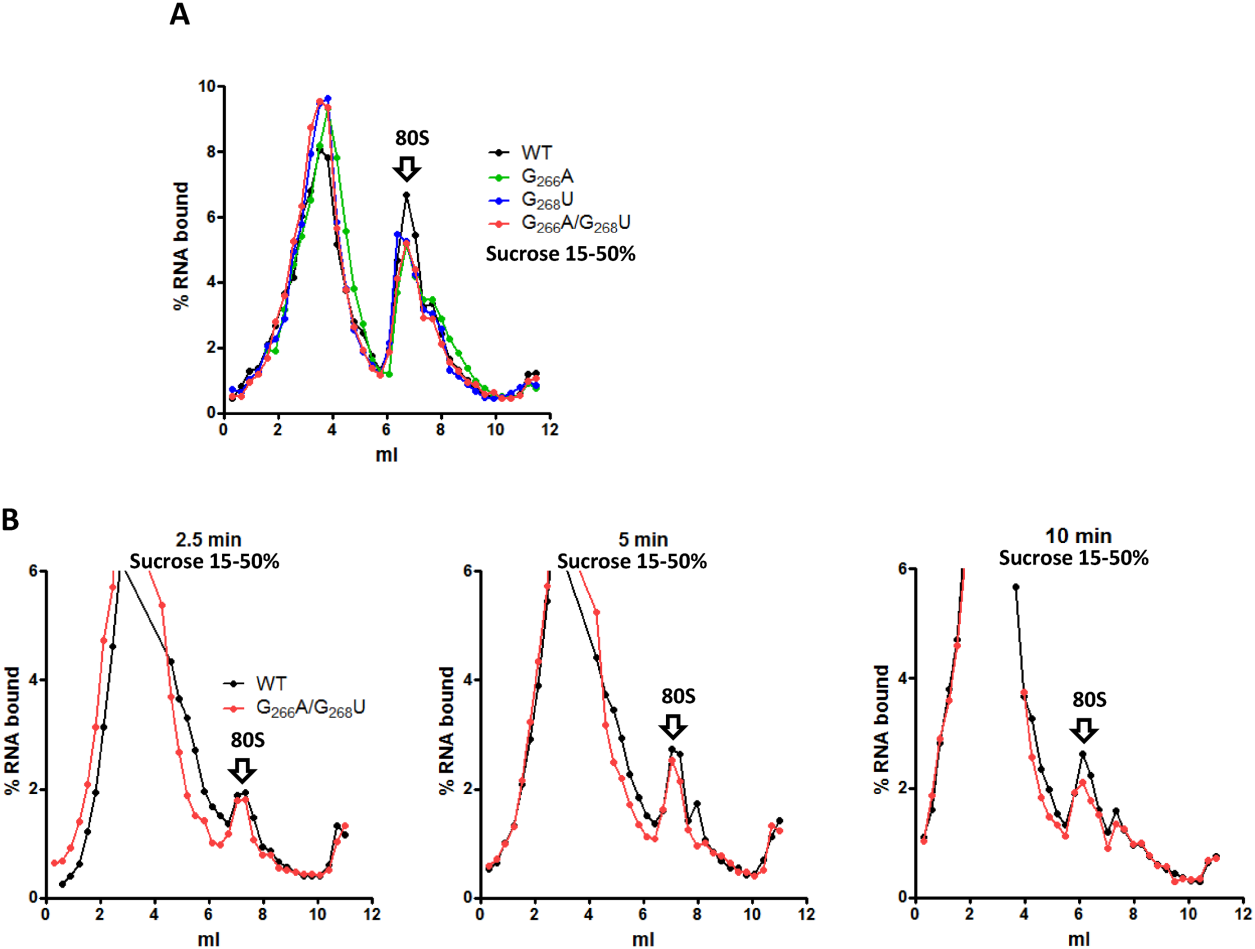
80S initiation complexes assembly on mutant and WT IRES. 2 pmol of WT (black) or G_266_A (blue) or G_268_U (green) or DM (G_266_A/G_268_U, red) ^32^P- labeled RNAs were used to program 50 μl of RRL and 80S complexes were resolved by fractionation onto 15–50% sucrose density gradients. RRL were treated with cycloheximide before to observe 80S accumulation (**A**) The WT and double mutant RNAs were incubated with untreated reticulocyte lysate and translation was stopped after 2.5; 5 or 10 min addition of cycloheximide (**B**) 0 ml is the top of the gradient (lighter fraction) while 12 ml is the bottom (heavier fraction). The results are representative of at least three independent experiments. Arrows indicating the 80S complex were positioned according to control experiments carried out with a cap-dependent gene and according to UV profile of gradients carried out in the same conditions with purified 40S, 60S and 80S complexes (Supplementary Figure S7). The small higher density peak corresponds to a disome due to some leakage in the cycloheximide blockage.

To gain further insights into the step of initiation affected by loop IIId mutations, we first evaluated the ability of mutant IRESes to form initiation complexes. To analyze the 80S initiation complexes paused on the initiation codon, the RRL was pretreated with cycloheximide, drug that inhibits translocation of the initiation complex, but not the preceding steps of the initiation process. Complexes assembled under such conditions were analyzed on a sucrose gradient. Surprisingly, the accumulation of 80S complex on the mutant IRESes under such conditions is only marginally lower than for the WT IRES (Figure 3A). Clearly, this slight difference does not explain the important effect of the mutations on translation. Next we evaluated whether the rate of initiation complex formation might be altered in the mutants by following complex accumulation over time. Here again, no major differences could be observed when the WT and the double mutant are compared except that the latter accumulates slightly less initiation complexes over the same period of time (Figure 3B). From these experiments we conclude that neither the amount of 80S complexes nor their rate of formation can account for the low translation efficiency induced by the mutations in loop IIId. Instead, we reasoned that the 80S complexes formed on the IRES mutants are most probably not functional. This prompted us to follow the assembly of initiation complexes assembly on these RNAs.

**Figure 4.**
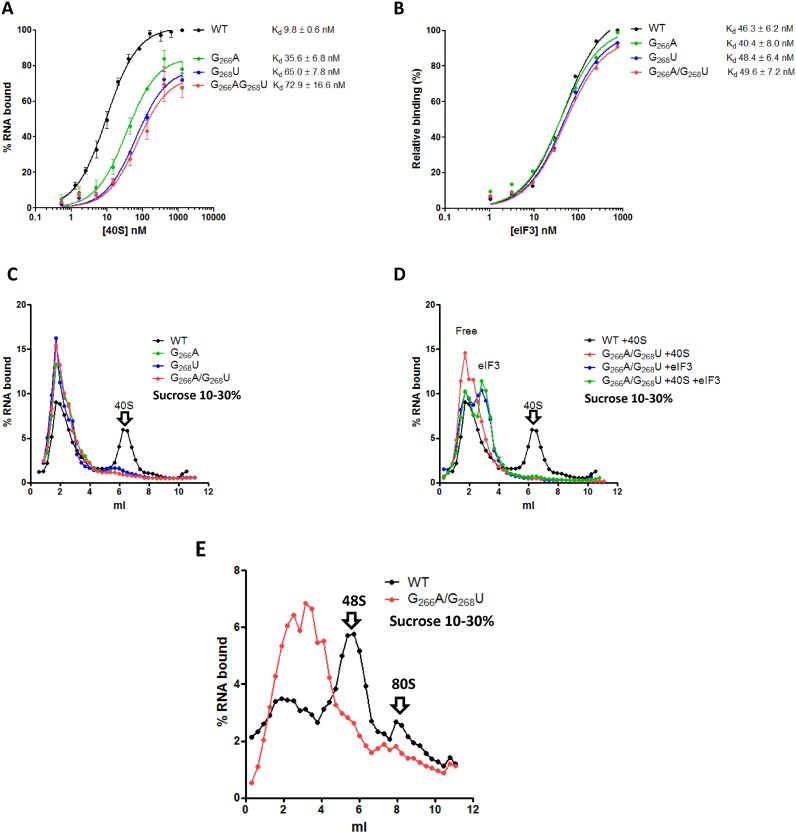
Loop IIId mutants interaction with ribosomal 40S and eIF3 and pre-initiation complex assembly. Binding curves of ^32^P labeled WT (black) or G_266_A (blue) or G_268_U (green) or DM (G_266_A/G_268_U, red) RNAs to purified 40S ribosomal subunits (**A**) or to purified eIF3 (**B**). The results are the mean of at least three independent experiments ± standard deviation. (**C**) Fractionation by 10–30% sucrose density gradient on which were run 20 nM of ^32^P labeled WT (black) or G_266_A (blue) or G_268_U (green) or DM (G_266_A/G_268_U, red) RNAs in the presence of 400 nM of purified 40S subunits. (**D**) Fractionation by 10–30% sucrose density gradient of 20 nM of ^32^P labeled WT in the presence of excess purified ribosomal 40S (400 nM) (black) or DM RNA (G_266_A/G_268_) in the presence of excess purified ribosomal 40S (400 nM) (red), or an excess of purified eIF3 (200 nM) (blue) or both (green). (**E**) pre-initiation complex assembly in RRL. ^32^P labeled WT (black) or DM (G_266_A/G_268_U, red) RNAs were incubated in RRL pre -treated with GMP-PNP. Complexes were fractionated on a 10–30% density gradients. The results are representative of at least three independent experiments. Arrows indicating the 40S subunit were positioned according to UV profiles.

Initiation complex formation on HCV IRES depends on its ability to form a complex with the 40S subunit of the ribosome, and to a lesser extent to directly bind eIF3. We therefore investigated the direct interaction of the mutant IRESes with these two components. As can be observed in Figure 4A, filter binding assays performed in the presence of purified 40S ribosomal subunits indicate that both the affinity and the maximal amount of complex formed are affected by the mutations in loop IIId. The increased *K*_d_ (*K*_d_ WT = 9.8 ± 0.6 nM; *K*_d_ DM = 72.9 ± 16.6 nM) is in agreement with the loss of a binding determinant and the lower ‘plateau’ further suggests that upon mutation fewer IRES molecules are capable of binding the 40S ribosomal subunit. It is of note that the G_268_U (*K*_d_ = 65 ± 7.8 nM)mutation seems slightly more detrimental than G_266_A (*K*_d_ = 25.6 ± 7.8 nM). Using the same approach, we monitored eIF3 binding to the IRES and observed that none of the mutants significantly disturbs eIF3/HCV IRES binding (Figure 4B). The 40S/HCV complex formation was then followed by sedimentation on sucrose gradient under conditions where most of the HCV IRES should be in complex with the 40S ribosome. In contrast with our filter binding assay results, only a very marginal amount of complex is detected with the single mutants, although we could clearly observe the WT IRES-40S complex (Figure 4C). We hypothesize that the hydrodynamic constraints applied during centrifugation could disrupt the complex if it is not compact enough. We therefore tried to stabilize the complex by increasing Mg^2+^ ions concentration to 6 mM (54), adding eIF3 and/or eIF2 ternary complex. Nevertheless, 40/43S complexes on the mutant RNAs were never observed (Figure 4D and Supplementary Figure S2). Finally we analyzed the 48S-like (33) complex formation by incubating WT or mutant RNAs with GMP-PNP-treated RRL. This non-hydrolysable analog of GTP precludes the formation of 80S complexes, thus inducing the accumulation of the 48S pre-initiation complex. Once again, and in sharp contrast to the WT IRES situation, we were unable to detect any high molecular weight complexes when the IRES mutants are used (Figure 4E). This result was unexpected because 80S initiation complexes directly derive from 48S-like pre-initiation complexes. This observation suggests a drastic effect of the mutations on an early step of the initiation complex assembly. Altogether our results indicate that the mutant IRES forms a non-functional complex with the 40S which is unstable on a sucrose gradient, but can proceed to assemble more stable but non-productive 80S particles. Alternatively the 80S complexes could result from the direct binding of the IRES on naked 80S as described *in vitro* (40,54). However this seems unlikely in cell extracts where the 80S complexes available are already engaged on mRNAs.

### Probing the HCV IRES structure in solution upon loop IIId mutation

**Figure 5.**
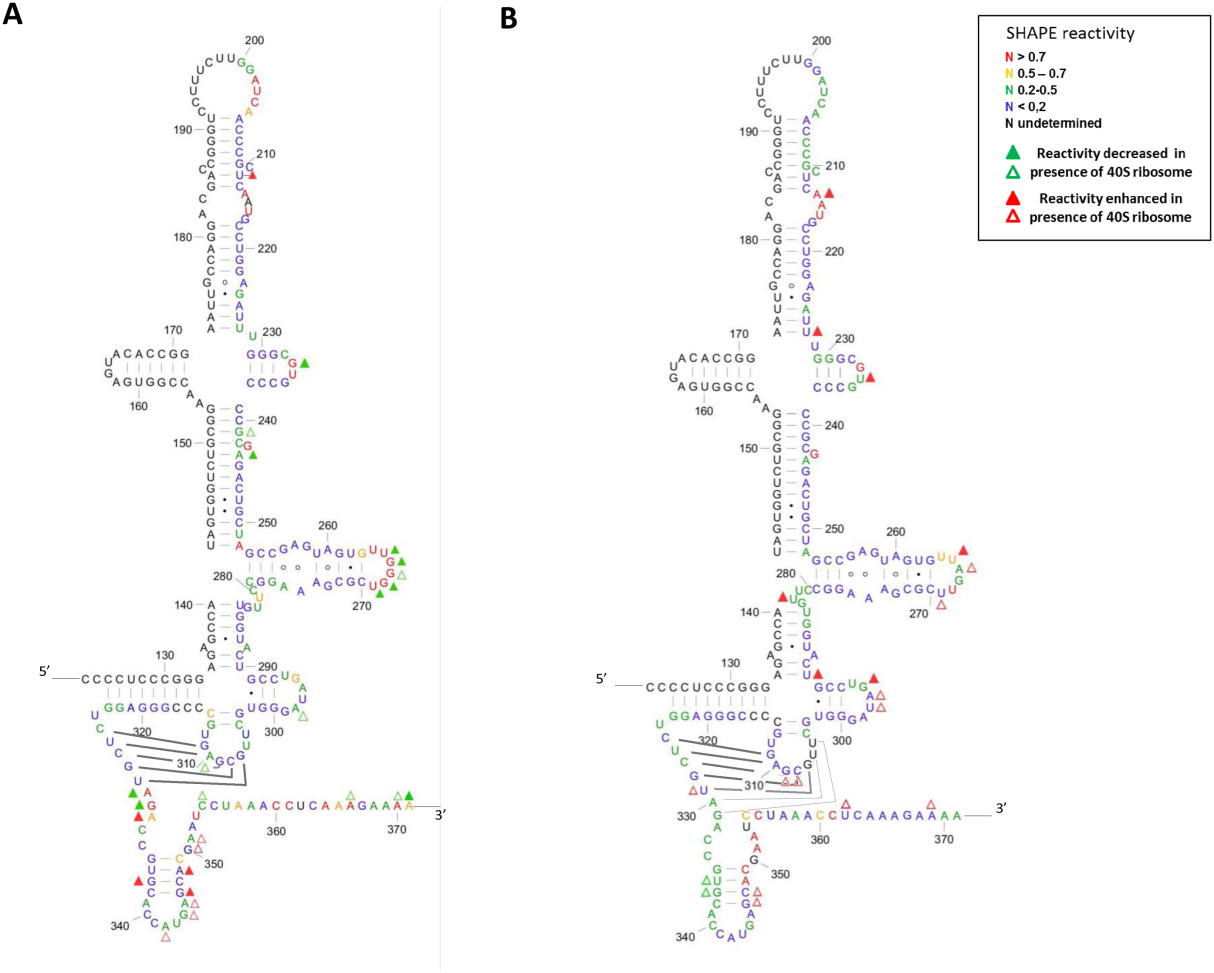
SHAPE reactivity of the WT (**A**) and the DM IRES (G_266_A/G_268_U) (**B**) in presence and absence of the 40S ribosomal subunit (400 nM). Relative reactivity calculated with QuSHAPE reported on the secondary structure of the WT and DM IRES. The nucleotides are numbered from the viral transcription initiation start. Only domains III and IV are represented, although the experiment was carried out on the Fluc construct containing domain II and the Fluc coding region (see Materials and Methods). The color code represents the reactivity of each nucleotide as specified in the box. Reactivity values are the mean value from five independent experiments for the WT and five for the DM, they were carried out using two different preparations of both 40S ribosomal subunit and IRESes RNA. Triangles represent footprints observed in presence of the ribosome. The green triangles represent positions for which the reactivity is decreased in presence of 40S (full triangle: *t*-test < 0.05, ratio *r* (without 40S/with 40S) >1.5, absolute value of the difference d (|without 40S – with 40S|) >0.1; hollow triangle *t* < 0.15, *r* > 2.25, *d* > 0.15). The red triangles marks positions where the reactivity is enhanced in presence of 40S ribosomal subunit (full triangle *t* < 0.05, *r* > 0.6, *d* > 0.1; hollow triangle *t* < 0.15, *r* > 0.4, *d* > 0.15). The base pair forming the pseudoknot are represented by solid lines, in the mutant the probing data does not exclude that the pseudoknot is extended by 2 base pairs represented as dotted lines. Figures were drawn using XRNA (http://rna.ucsc.edu/rnacenter/xrna/xrna.html).

RNA structure was probed using the high-throughput SHAPE technology using 1-methyl,7-nitroisatoic anhydride (1M7) as a probe for ribose flexibility (51). Briefly, RNA is incubated with 1M7 which reacts with flexible ribose moieties. Such modification induces a premature elongation stop upon reverse transcription of the RNA, allowing the mapping of reactive sites within an RNA by comparing the elongation profiles obtained with 1M7-treated RNA to a mock sample. WT, G_266_A, G_268_U and the double mutant G_266_A/G_268_U were subjected to the SHAPE process. Due to the experimental setup, data could only be analyzed from G_200_ to A_371_, where most of the ribosome binding determinants lie on the HCV IRES (34–36,55). The 1M7 reactivity profile obtained with the WT IRES construct is in very good agreement with the currently accepted secondary structure model and with what is known about the 3D structure. Most nucleotides modeled in Watson-Crick helices, or as non-canonical base pairs are poorly reactive to 1M7 (Figure 5A and Supplementary Figure S2). Noticeable exceptions with high reactivites are A_252_ participating in the first base pair of the main stem from domain III, A_330_, which is very reactive but often considered as part of the pseudoknot and _331_GA_332_, although they are often shown base paired to _353_UC_354_. Consistently U_353_ is also highly reactive, indicating that the first two base pairs of domain IV are not formed under the conditions tested. In contrast, _333_CC_334_, _350_GAA_352_ and domain IV apical loop (_339_ACCAUGA_345_), are poorly reactive to 1M7. Interestingly in this study, and in agreement with all structure probing data to date, loop IIId is very reactive to 1M7. The mutants were subjected to the same treatment and they all show different modification patterns (Figure 5 and Supplementary Figure S3B–G). WT and mutant RNA probing experiments were performed at least in triplicate, the reactivity of a given nucleotide in two different constructs was compared using statistical and arithmetical criteria (see Materials and Methods). As expected, the reactivity of the IIId domain loop is altered in all mutants, however not to the same extent. The G_266_A mutation has the most drastic effect, as all nucleotides within loop IIId but G_268_, become mostly unreactive (Supplementary Figure S3B and S3E). We also observed a slight reactivity decrease of _241_GC_242_ and A_244_. These nucleotides surrounding the bulged G_243_ are embedded in the stable stem III3 and are weakly reactive in the WT construct. We interpret this result has an entropic effect of the stabilization of loop IIId. More importantly, a very strong effect is observed in the domain IV region. The residues _330_AG_331_, which are very reactive in the WT IRES become poorly reactive in the mutant, conversely _351_AA_352_ become very reactive (Supplementary Figure S3B and S3E). The G_268_U mutation has a modest local and long distance effect (Supplementary Figure S3C and S3F). The mutated position remains very reactive, and while _266_GG_267_ are significantly less reactive than in the WT RNA, they remain very flexible. Few other changes are observed, _291_GCC_293_, U_297_ in domain IIIe and the two G-C base pairs closing helix IV are slightly more reactive. Overall, all the changes observed upon G_268_U mutation present weak amplitudes, and the structure of this mutant is likely to be very similar to the wild type (Supplementary Figure S3C and S3F). The double mutant is rather similar to the G_266_A single mutant (Figure 5, Supplementary Figure S3D and S3G). We observed a reactivity drop for all nucleotides within loop IIId. G_268_ remains reactive but positions 266 and 267 are only moderately reactive. We also observe a slight but significant stabilization at the junction between III3 and III4, but the bulged G_243_ remains reactive. Similarly to what is observed upon G_266_ mutation and in contrast with the WT IRES, positions _330_AGA_332_ are very poorly reactive to 1M7.

### Footprint analysis of the 40S/IRES RNA interaction

The same experiments were repeated in the presence of ribosomes, and the pattern differences (footprints) were evaluated (See materials and methods). A 40S ribosomal subunit concentration of 300 nM was used, in order to reach binding saturation even for the mutant constructs. As previously described, using RNase T1 or another SHAPE reagent (NMIA), the most obvious footprint induced by ribosome binding on the WT IRES occurs in loop IIId (35,55) (Figure 5A). One should note that these nucleotides remain significantly reactive, and this is especially true for G_268_ which remains one of the most reactive nucleotide of the IRES (Supplementary Figure S3A). We also observed protections in loops IIIc and IIIe which are part of the minimal ribosome binding site and are in close contact with the ribosome as observed by cryoEM (11,40). Positions G_241_ and A_244_ are less reactive, although this side of the helix is not in contact with the ribosome. We interpret this result as a stabilization of this paired region surrounding a bulged nucleotide. Finally, _330_AG_331_ which are very reactive in the isolated IRES are essentially inert in presence of the ribosome. This could either result from direct contacts with the ribosome or from intramolecular interactions owing to the extension of the pseudoknot. In contrast, we observed an increase of reactivity for many nucleotides within domain IV which strongly suggest its destabilization. Unexpected increases in reactivity were observed outside the 40S ribosomal subunit minimal binding site at U_212_, supported by the modification of the RNase cleavage pattern in this region (35), and are unlikely to reveal any direct contact. In the case of the G_266_A mutant four protections were observed, two in domain IIIc (G_229_ and G_235_), one in helix III3 (G_246_) and one at the bottom of IIId (G_253_) (Supplementary Figures S3B and S4A). Importantly, the characteristic protections observed for the WT IRES upon ribosome binding, i.e in loop IIId and for _330_AG_331_, are not observed in the G_266_A mutant. Nonetheless, as these sequences are poorly reactive in the isolated mutant IRES it is difficult to conclude on their potential status change. We observed few chemical reactivity enhancements in domain IV, although most nucleotides in this region remain moderately reactive. Noteworthy, we also observed a strong increase of reactivity for few nucleotides in helix IIIb (Supplementary Figures S3B and S4A). In the case of the G_268_U mutant, no nucleotide in loop IIId is protected from modification upon 40S ribosomal subunit binding (Supplementary Figures S3C and S4B). In contrast, we observed slight but significant protections scattered from domains IIId, IIIe and IIIf, up to the PK, which have been shown to form a compact four-way junction stabilized by the two pseudoknots closures (56). Our results suggest an overall stabilization of this tertiary structure by the 40S subunit of the ribosome. This is in clear contrast to the double mutant for which almost no reactivity decrease upon ribosome binding was detected, except for residues 336–337 in the helical portion of domain IV. In addition, nucleotides in loops IIIc (U_235_), IIId (U_265_, A_266_, U_269_), IIIe (G_295_-U_297_), and in domain IV (G_346_-C_347_) appear to be more reactive in presence of the 40S ribosomal subunit (Figure 5 and Supplementary Figure S3D). Although we have no explanatory model for these observations, they strongly suggest that the ribosome is bound in an incorrect way. Overall, although the ribosome binds the mutant IRESes, the footprint observed on the mutated IRES RNAs strongly suggest that the latter do not make the same contacts and therefore are incorrectly positioned.

**Figure 6.**
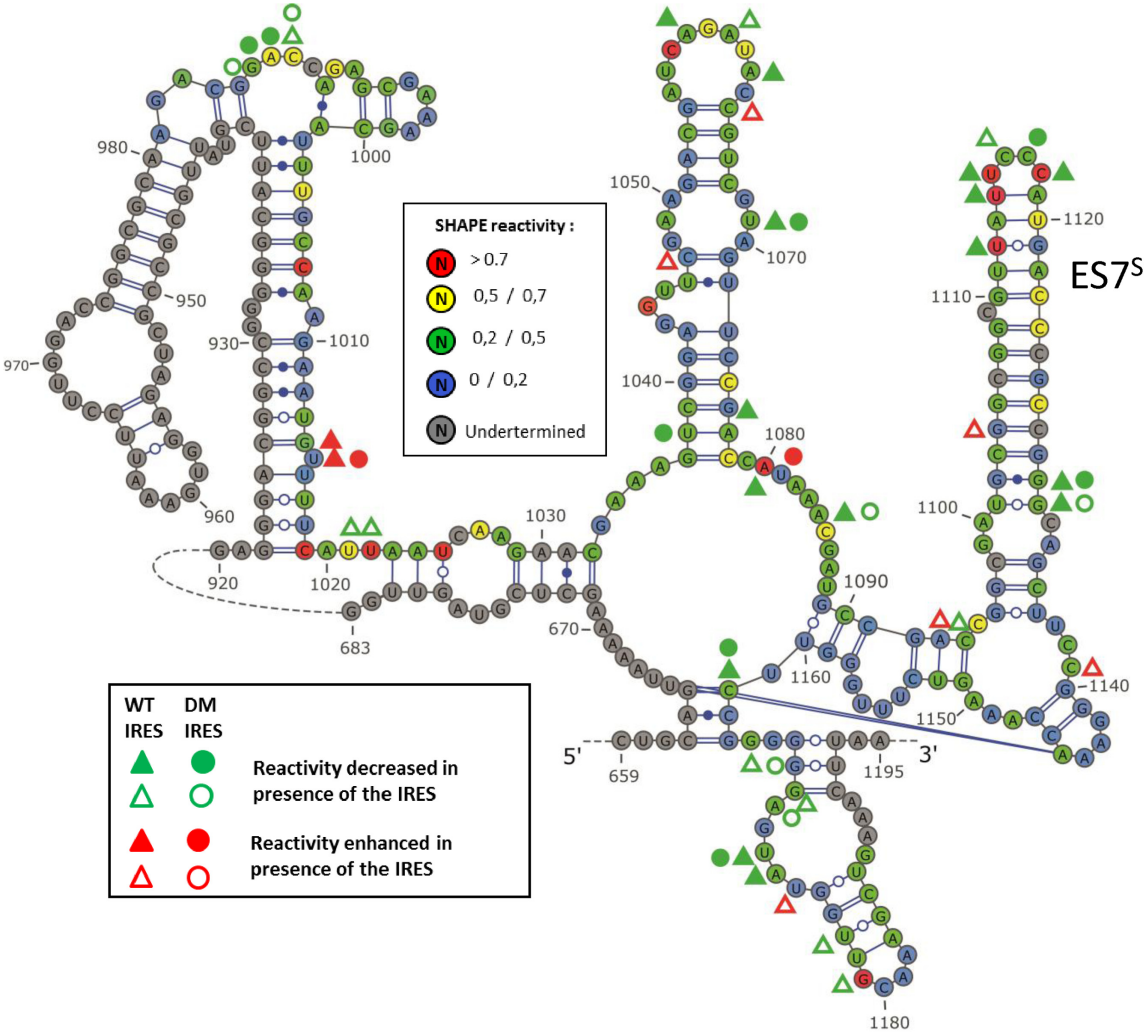
SHAPE reactivity of the 18S rRNA in presence or absence of WT or DM IRES (300 nM). The scheme represents the secondary structure of the 18S rRNA from 659 to 1195, the portion from 683 to 920 has been omitted, ES7^S^ is indicated. The shape value is color-coded as indicated in the box. Reactivity values are the mean value from three independent experiments that were carried out using two different preparations of both 40S ribosomal subunit and IRESes RNA. Triangles represent footprints observed in the presence of the WT IRES, while circles are footprints observed in the presence of the DM IRES. The decreased and enhanced reactivity in presence of the WT HCV IRES and the DM HCV IRES are marked with triangles and circles respectively. Green symbols are positions protected in presence of the IRES (full *t* < 0.05, *r* > 1.5, *d* > 0.1; hollow *t* < 0.15, *r* > 2.25, *d* > 0.15) and red symbols note positions where the reactivity is enhanced (full *t* < 0.05, *r* > 0.6, *d* > 0.1; hollow *t* < 0.15, *r* > 0.4, *d* > 0.15). The figure was drawn with VARNA (81).

**Figure 7.**
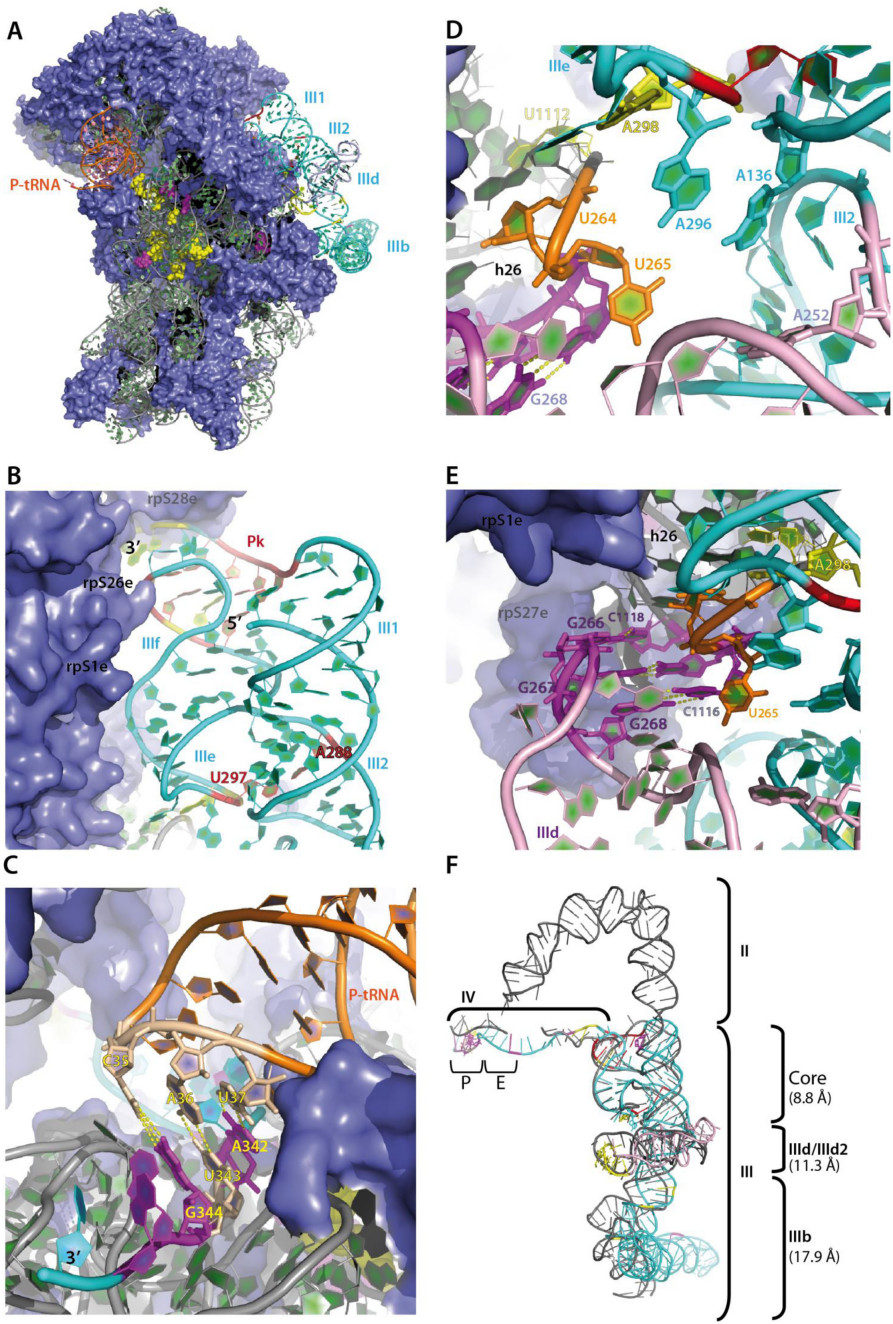
Three-dimensional whole atom model of the HCV IRES. (**A**) General view of the model showing the IRES RNA (cyan) bound on the platform side of the 40S subunit. Domain IIId appears in light blue. SHAPE protections (yellow) and enhancements (purple) are mapped on the ribosomal RNA as spheres and on the IRES RNA as ribbon (grey or cyan, respectively). Ribosomal proteins are depicted as slate surface. (B–E) Close-up on four regions important for the binding of the IRES to the 40S subunit. (**B**) Depiction of the region comprising the two pseudoknots (red ribbon: (Pk-IIIf _307_GG_311–325_CU_329_ and III_2_-IIIe _297_U-A_288_)) locking the 4-way junction in active conformation. The tethering point of the IRES domain II, which is not presented in this model, is indicated by the label 5′, while the label 3′ indicates the inlet of the mRNA channel in between ribosomal proteins rps26e and rps28e (in the background). Ribosomal proteins forming the binding interface with the IRES are indicated. (**C**) Depiction of the P site codon-anticodon interaction resulting from unwinding nucleotide by nucleotide the IRES domain IV (cyan ribbon) following the path of the mRNA in the S. cerevisiae ribosome (61). The P site tRNA (orange ribbon) contacts the IRES AUG on which enhancements to 1M7 are depicted in purple, indicating that the P site residues become exposed upon 40S binding. Residue numbers are indicated. (**D**) A detailed view of the A pocket resulting from the IRES folding. Residues A_136_, A_252_, A_296_ and A_298_ build up the pocket. This conformation is supported by close proximity of residues A_298_ and U_1112_ at about 6 Å one from another. Consequently A_136_ and A_296_ lie in very close distance from U_264_ and U_265_ (orange sticks). These residues were observed to form two unanticipated A-U base pairs in the cryo EM structure of the 40S-bound HCV IRES (40). (**E**) Model of the IIId domain interaction with the apical loop from the ribosomal h26 (purple). Numbers of the involved residues are indicated. Parts of the IRES core are visible on the right side (cyan ribbon and the IIIe pseudoknot). Helix h26 (grey) is in the background and points from upwards while IIId points from downwards. U_264_ and U_265_ (orange sticks) are again displayed. (**F**) Comparison of our model with the cryo EM structure of the 40S-bound HCV IRES (40). Rmsd values for individual regions of the IRES calculated with chimera (45) following whole superimposition are indicated. The core of the IRES up to the IIId domain presents values compatible with the correct identification of global architectural features and of the overall molecular topology. Higher values observed for the IIIb domain result from less well defined conformation as shown in the cryo EM structure in which no density is observed for the tip of IIIb. SHAPE protections (yellow) and enhancements (purple) are mapped on the IRES RNA as ribbon.

Similarly, the 18S rRNA structure within the 40S subunit was probed with 1M7 in absence or presence of a saturating IRES concentration. The WT and double mutant IRES footprints were detected in the region surrounding ES7^S^ loop (nucleotides 981–1187), which base pairs with loop IIId. The reactivity profiles (Figure 6 and Supplementary Figure S5) are in good agreement with the established secondary and tertiary structures, although some regions appear more flexible than anticipated. These are often nucleotides predicted to be involved in Watson-Crick base-pairs, albeit located at the edge of a bulge, such as C_1006_. As expected, the WT IRES strongly protects _1116_CCC_1118_ while the double mutant only marginally protects C_1117_. Nucleotides U_1112_ and U_1114_ closing the ES7^S^ loop which are unexpectedly reactive in absence of IRES are also strongly protected upon WT IRES binding. More surprisingly, we observed many modification pattern alterations at a distance from ES7^S^ when the WT IRES is bound to the ribosome. Mapping of these alterations on a 3D model of the HCV IRES - ribosome complex derived from the cryo-EM structure of the CSFV IRES bound to the rabbit ribosome (11) reveals that most of the positions involved are buried within the ribosome structure (Figure 7). Such reactivity enhancement cannot result from direct contacts of these nucleotides with the IRES, and probably reflects subtle structural rearrangements (see also (57)). Such reactivity alterations were also observed with the double mutant IRES, but only for a subset of position (C_988_, C_1162_, U_1069_, U_1172_, G_1130_ and G_1131_). In addition few changes appear to be specific for the double mutant IRES (G_985_, G_986_, A_987_, U_1081_ or A_1170_)(Figure 6 and Supplementary Figure S5).

### Whole atom modeling of the IRES–40S subunit complex

In order to generate a three-dimensional (3D) framework to visualize our data, a whole atom model was built by taking the cryo EM structure of the CSFV IRES bound to the 40S subunit and eIF3 (11) as a template. We used this structure as the latest cryo EM structure of the 40S-bound HCV IRES (40) was published after we generated the present model. In the 40S-bound CSFV structure, the pre-initiation state is closer to our system than the initiation state with the 80S already formed as presented in (26). The high structural homology between the IRES RNAs of CSFV and HCV together with the help of available NMR or crystal structures of the HCV IRES subdomains allowed us to model with confidence the structural units from domain III (Figure 7A and Supplementary Figure S6A), i.e. the core 4-way junction including stems III1, III2 and loops IIIe, IIIf and the pseudoknot; the 3-way junction containing stems III2, III3 and loop IIId; and finally the apical 4-way junction encompassing stem III3, and loops IIIa, IIIb and IIIc. Note that loop IIIb is responsible for eIF3 binding. The core 4-way junction containing the pseudoknots was modeled as in (56), and the relative position of the five helices was refined using the 40S-bound CSFV structure as a template. Despite slight displacement of the helices, the interactions leading to the simultaneous formation of the two pseudoknots (PK1: G_307_CGAG_311_-C_325_UCGU_329_, and PK2: A_288_-U_297_) were preserved (Figure 7B). The 3-way junction was then tethered to the core 4-way junction. The geometrical constraints of the model naturally oriented loop IIId toward h26 in ES7^s^. A loop E motif was integrated in domain IIId (Supplementary Figure S6B) as suggested earlier (58) and further observed in solution (36) using the sarcin loop as a template (59). The apical 4-way junction was then tethered to the 3-way junction. Two coaxial stacks were built, one consisting of the IIIc hairpin onto the stem joining it to IIId, and hairpins IIIa onto IIIb (Not shown). Non-canonical interactions were based on the solution structure of loop IIIb in isolation (60), although the structure of the domain may be different in the 40S–eIF3-bound context. The refined model presented a satisfactory geometry with the different domains occupying space regions compatible with the 40S-bound CSFV cryo-EM structure. The HCV IRES model presents the first nucleotide after the IIIf pseudoknot at the same position as the CSFV IRES RNA. Domain IV was treated as a single-stranded RNA, and fit into the mRNA channel taking advantage of the cryo EM structure of the *Saccharomyces cerevisiae* ribosome bound to the mRNA and the initiator P site tRNA (61). Unwinding domain IV nucleotide by nucleotide following the mRNA track brought the AUG start codon exactly into the P site (Figure 7C).

To model the human ribosome, the *T. thermophila* (T.th) 40S subunit (44) was first docked in the rabbit cryo EM map (11). In a second step, we changed the sequence of the T.th ribosomal helix h26 to *humanize* it. The human h26 is 4 base pairs longer than the T.th, but the human apical tetraloop is 3 nt shorter than the T.th. The h26 apical loop was modeled as the L5 tetraloop in the *Azoarcus* group I intron (62) in order to adopt a pseudo-helical conformation starting at the second residue. The extension of h26 generates a clash with loop IIId. We reasoned that, (i) the position of the IIId domain may result from incorrect secondary structure at the 3-way junction interface, and that (ii) remodeling of some interactions may bring the IIId domain structurally closer to the CSFV IRES domains IIId_1_ and IIId_2_. Consequently, U_141_–A_252_, A_142_–U_251_ and the first base pair of IIId (G_253_–C_279_) were broken, and C_279_ was paired to G_283_ in a WC *trans* geometry (63). The same geometry was adopted for base pairing U_141_ to U_251_. These modifications of the secondary structure promote stacking continuity between the helical segments and allow building a pseudo-CSFV IIId_2_ loop (Supplementary Figure S6B). The optimization of the model consistent with the SHAPE reactivities allows the placement of the IIId loop at a place compatible with the formation of the kissing interaction of the three G residues from loop IIId with the three C residues in the humanized h26.

The resulting model presents a cleft accommodating the tip of h26. The walls of this cleft are made from the apical loops IIId and IIIe, in agreement with SHAPE analysis, which points to a potential interaction between A_298_ from IIIe with U_1112_ from h26 (Figure 7D). Although the role of loop IIId was demonstrated earlier, the model interestingly suggests the spatial convergence of four A residues, namely A_136_, A_252_, A_296_, and A_298_, in the immediate vicinity of h26 (Figure 7D). This phenomenon could be specifically involved in stabilizing the IRES/40S interaction.

## DISCUSSION

### Loop IIId mutations not only alter the local structure, but the IRES global folding

Because of their conservation, the three guanines in loop IIId have been detected in very early studies as important type III IRES functional determinants (30,32,35). In most cases they have been mutated to three cytosines and the consequences of their mutation on the IRES structure has been evaluated by monitoring 48S assembly (64) and RNaseT1 probing (31). Here, we took advantage of the high-throughput SHAPE technology to probe the flexibility of every nucleotide from G_200_ to A_371_ for the WT IRES and the three loop IIId mutants. The results observed with the WT IRES are in very good agreement with previous data obtained using various probes (31,35,55,65–70) and with the currently accepted secondary structure model (65,66,71,72). However, 1M7 probing on HCV IRES in solution reveals several details that can be used to refine the model. First, A_330_ appears to be very reactive, consistent with the observation that it does not participate in the pseudoknot in the crystal structure of the IRES core 4-way junction (56). Conversely, nucleotides 307–311 and 325–329, which are involved in the pseudoknot, are not reactive. These data support the idea that the pseudoknot does not extend to U_306_–A_330_ and U_305_–G_331_ neither in solution nor upon complex formation with the small ribosomal subunit. Moreover, as deduced from the present model and the CSFV cryo EM structure (11), extension of the pseudoknot would result in overwinding the region upstream from domain IV, preventing the single-strand domain IV from engaging smoothly within the mRNA channel. Second, residues G_331_, A_332_ and U_353_ are very reactive in the free IRES, which is in opposition with their postulated base pairing together with C_354_. In contrast, residues within the domain IV loop containing the initiation codon appears to be poorly reactive. This is unexpected because the AUG triplet has been previously found to be targeted by another SHAPE reagent (The N-Methyl Isotaic Acid) (55,68), and G_344_ is sensitive to RNase T1 (35). However, it has been reported that 1M7 and NMIA do not exhibit identical reactivities to RNA flexibility, and that positions involved in non-canonical base pairs or long range interactions are often found reactive to NMIA while they are mostly unreactive to 1M7 (73). We thus propose that domain IV apical hairpin harbors some structural features preventing 1M7 targeting of nucleotides from the loop. In line with the poor stability of the domain IV hairpin, the binding of the 40S may contribute to unwind the 3′ end of the IRES so that it adapts to the mRNA cleft of the ribosomal subunit. Our model strongly supports this mechanism since unwinding following the mRNA tract (61) precisely positions the AUG codon in the P site.

As monitored using different structure probes in solution, loop IIId is very flexible strongly suggesting that it is neither structured, nor in interaction with another part of the IRES (31,35,55,65,66,68,69,74). Mutation G_266_A has a strong local effect since all loop IIId nucleotides become poorly reactive, except for G_268_. This mutation appears to have long-range effects. Most strikingly, _330_AG_331_ and G_243_ become almost inert while _351_AA_352_ become very reactive. The G_268_U mutation appears to have a milder effect, while the double mutant shows an intermediate pattern. These perturbations could be explained by rigidification of alternative conformations of the structure of the IRES in solution. This would explain why a smaller proportion of the mutants are able to bind ribosomes. Thus, the first effect of loop IIId mutations is an unexpected interference with the IRES global folding.

### Loop IIId mutants make improper complexes with the small ribosomal subunit

Loop IIId mutants are impaired for ribosome binding, and most studies relating the effects of these mutations concluded that the reduced affinity was responsible for the translational defect. However, the double mutant that is most defective in translation still retains a significant affinity for the 40S ribosomal subunit (*K*_d_ = 74 nM Figure 4). This can be explained by the observation that the 40S/IRES complex presents a large surface of interaction with the 40S (11,25,40), which does not only rely on the ES7^s^/domain IIId kissing-loop interaction. Total ribosome concentration in HeLa cells extract has been reported to be in between 0.6 and 1 μM (75,76), and we have estimated the free 40S subunits (or 43S particles) concentration in flexi-rabbit RRL (Promega) to be around 400 nM (Supplementary Figure S7). Such concentrations are five to tenfold above the *K*_d_ of the most affected mutant and therefore should not be a limiting factor. This is further supported by the observation that 80S complexes are assembled on the mutants with the same efficiency as on the WT IRES. Finally, if complex formation with IRES mutants were quantitatively affected, translation efficiency should be rescued by increasing the mRNA concentration used to program the RRL, which is not the case. The main indication that the mutant IRES/40S complexes have a qualitative defect comes from the density gradients analysis. The IRES RNA and the 40S ribosomal subunit were mixed in concentrations in which almost 100% of the RNA should be in complex with the ribosome. However only a marginal quantity of the IRES single mutants runs as a 40S complex when separated on a sucrose density gradient. Of note, less than 100% of the WT IRES runs as a 40S complex reflecting that this technique is not quantitative. The mutant IRES/40S complexes thus appear to be unstable on density gradients. These complexes were not stabilized by the addition of eIF3 or the ternary complex (eIF2-GTP, tRNA_Met_^i^) or when they were assembled in RRL in presence of GMP-PNP. This may reflect the fact that complexes formed with the mutants are less compact, less hydrodynamic, therefore promoting their disassembly along the gradient. Alternatively, the binding defect may be accompanied by an increase of the exchange rate if the IRES mutants are unable to promote a structural rearrangement stabilizing the complex upon binding to the 40S. Cryo-EM studies reveal a conformational change of the 40S ribosomal subunit induced by the presence of the WT IRES (11,25,77). We used the SHAPE technology to probe the contact sites and the conformational rearrangement of both the IRES and the 18S ribosomal RNA. To our knowledge, this is the first time that protections are shown on both the IRES and the 18S rRNA in the same study, and moreover that the footprint in ES7^S^ is shown to disappear upon mutations in loop IIId. With the WT construct, we observed a strong reactivity decrease in both the 18S ES7^S^ and the loop IIId. This further supports the kissing loop interaction first proposed by Malygin *et al*. (38) and recently functionally demonstrated (39). The protection of _266_GGG_268_ in loop IIId from attacks by nuclease or chemical agent has been observed several times (31,35,36,55). Similarly, _1117_CC_1118_ in ES7^S^ have been shown to be protected from DMS and hydroxyl-radical modification upon binding of the HCV wild type IRES. Our model led to the conclusion that the interaction between loop IIId and the ribosomal h26 from ES7^S^ is reinforced by loop IIIe. That is, A_298_, presents its ribose moiety in the vicinity of U_1112_. In addition, the architecture of the core 4-way junction encompassing IIIe concentrates four adenine residues (A_136_, A_252_, A_296_ and A_298_) in the immediate vicinity of h26 and are likely to contribute additional contacts. Some specific contacts involving these adenines were indeed observed in the recent cryo EM structure of the 40S-bound HCV IRES: A_136_–U_1115_ and A_296_–U_1114_ (40). Although our SHAPE data did not suggest these interactions directly, they detected the nearby contact between A_298_ and U_1112_, which then pointed to the potential contacts between the A pocket adenines and the U residues on the 5′ side of loop IIIe (Figure 7D). In the absence of SHAPE data supporting these potential interactions we did not provide any structural model for these additional interactions. However our modeling work correctly identified the IRES residues involved, and our probing data are consistent with the contacts modeled. The interaction A_298_–U_1112_ predicted from modeling and probing data is possible in the cryoEM model although it has not been specified. Our model also predicts that the architecture of the 3-way junction encompassing loop IIId, which forms a one-base pair stem-loop (C_279_–G_283_) stacking on the stem IIId, leads to the formation of an additional structural element resembling loop IIId2 from the CSFV IRES. Such an element was indeed observed in the 40S-bound cryo EM structure of the HCV IRES (40). However, C_280_ instead of C_279_ in our model, was found paired to G_283_. The presence of such an unforeseen element in the HCV IRES indicates higher order structural organization within IRES families. In addition we observed the concomitant reactivity decrease of _330_AG_331_ and increase of most residues within domain IV upon 40S subunit binding. This indicates that upon IRES binding to the ribosome, _330_AG_331_ establish contacts with the ribosome, while domain IV is destabilized to be accommodated in the ribosomal mRNA channel. The 3D model presented in this study supports this interpretation. Indeed A_331_ and G_332_ are in close proximity with ribosomal proteins S26e and S28e (Figure 7C). This is in agreement with recent cryo EM structures (26,40) and with previous studies showing that an over-stabilized domain IV no longer supports translation (67). In addition, the pseudoknot length is important for positioning the AUG codon in the P site (72). Interestingly, in the double and G_266_A mutants, A_331_ and G_332_ appear poorly reactive prior to ribosome binding and are not further protected upon 40S ribosomal subunit binding. The extension of the pseudoknot in these mutants would hinder binding with the ribosome. The distance between the A residues from the AUG start codon and the inlet of the mRNA channel between ribosomal proteins S26e and S28e may be used as a molecular ruler to identify the 3′ last nucleotide involved in the pseudoknot in IRESes related to HCV.

When mapped on the structure model of the WT complex, it appears likely that other reactivity decreases, such as those observed for A_298_ or G_233_ could reflect contact points between the IRES and the ribosome. In contrast, the decreased flexibility of G_241_ and A_244_, which are located on opposite sides of the helix with both facing the solvent, are interpreted as a global stabilization of the IRES structure upon 40S binding. Finally, some residues of the WT IRES like A_252_, within the 3-way junction encompassing loop IIId also appear flexible in 40S bound and unbound context. This observation guided us to locally modify the secondary structure to improve the contact between loop IIId and the tip of h26. In the cryo EM structure A_252_ has been modeled paired to U_141_, although the helices in this region appears unusually stretched and the reactivity of A_252_ is incompatible with a stable pairing (40). It is thus possible that the CSFV IRES recognizes the 40S subunit in a manner slightly distinct from the HCV IRES in this region. Alternatively the cryoEM image maybe less well defined in this region. SHAPE reactivity modifications are also observed with the IRES mutants upon incubation with the 40S ribosomal subunit, confirming that these mutations do not prevent complex formation. The ribosome footprints on the IRES appear distinct from one another and from the WT IRES. These footprint patterns are difficult to compare because the reactivity profiles of the corresponding naked RNAs are also distinct. As expected, none of the mutants show a reactivity decrease in loop IIId in the presence of the 40S ribosome subunit. For the G_266_A RNA, the main footprints reflect the exposure of domain IV. In contrast, most modifications observed for the G_268_U RNA could reflect the stabilization of the tertiary structure of the ribosome binding domain (domain IIIdef, the pseudoknot and domain IV) preventing the destabilization of domain IV and possibly its accommodation in the mRNA channel of the ribosome. Finally, in the double mutant, most pattern modifications upon ribosome binding reveal an enhanced reactivity essentially in loops IIId and IIIe indicating loss of the synergy between the different key interactions between IIId, IIIe and h26. The reactivity pattern changes in the 18S rRNA upon WT IRES binding were also mapped on the 3D structure of the ribosome. All the flexibility changes reside in regions buried within the ribosome, hence not accessible to the IRES, except for nucleotides within ES7^S^. Such footprints are likely to result from conformational rearrangements of the ribosomal 40S driven by binding of the IRES. As the 40S ribosomal subunits appear somehow flexible, the observed structural rearrangements could correspond to the IRES-induced stabilization of an initiation-prone conformation naturally adopted by the 40S subunit.

Fewer footprints are induced by the double mutant IRES. Several of them appear to be specific of the mutant IRES, and most of them appear to be a limited version of what is observed with the WT IRES. No change in reactivity is observed in ES7^S^ loop, except for a weak protection of C_1116_. However, our footprint and filter binding assay data show that loop IIId mutants still bind the 40S subunit of the ribosome. The associated reduced affinity may indicate a recognition mode of the ribosome by the IRES mutant unable to induce the 40S conformational rearrangement necessary to promote translation initiation. Although we cannot rule out that the 80S complexes observed with the mutant result from the direct binding of the IRES to naked 80S ribosome, it seems unlikely because 80S complexes observed in cell extracts are already engaged on an mRNA. It is therefore more likely that 48S-like altered complexes can nevertheless quantitatively recruit the 60S subunit, although the resulting 80S complexes are translationally impaired. This is reminiscent of domain II mutants that can recruit initiation complexes but are unable to properly manipulate the ribosome to trigger translation (23). Indeed a recent cryo EM derived HCV IRES–80S complex model shows that the HCV IRES induces a 40S/60S relative rotation resembling a translocation movement (26). Our data and 3D model show that loop IIId and IIIe act as a tweezer grabbing helix h26 in the 18S rRNA through specific base pairing. We posit that this is essential to trigger the conformational changes of the ribosome necessary to promote translation. Interestingly, loop IIId is also the site of a long distance interaction with a sequence further downstream in the viral genome (69,78), and the site of interaction with the core viral protein (79). These mutually exclusive interactions are likely to be important to regulate the virus life cycle. The recently published high resolution model is derived from the cryoEM images of a 80S-HCV IRES complex to which the 60S densities have been subtracted (40). The convergence of the two models validates our approach and gives confidence in the conclusions drawn independently using radically different methodologies. On the one hand, this confirms that the cryo EM model derived from 80S-HCV IRES complex is valid for 40S-HCV IRES in solution. On the other hand, it exemplifies once more the sensitivity and the robustness of the SHAPE technology to analyze RNA molecular contacts. In several instances, our conclusions highlight the importance of the tertiary and secondary structure co-adaptation upon binding of the 40S. This phenomenon first described for the P5abc domain of Tetrahymena group I intron (80) is likely to be an important trend of RNA folding and interaction.

## Supplementary Material

SUPPLEMENTARY DATA
