# Supplementary material for: LOOP IIId of the HCV IRES is essential for the structural rearrangement of the 40S-HCV IRES complex
Source: Nucleic Acids Res. 2015 Nov 30;44(3):1309–25. doi: 10.1093/nar/gkv1325 (PMC4756818; doi:10.1093/nar/gkv1325)
Supplement: SUPPLEMENTARY DATA [file supp_44_3_1309__index.html]

LOOP IIId of the HCV IRES is essential for the structural rearrangement of the 40S-HCV IRES complex — LOOP IIId of the HCV IRES is essential for the structural rearrangement of the 40S-HCV IRES complex — SUPPLEMENTARY DATA 

# LOOP IIId of the HCV IRES is essential for the structural rearrangement of the 40S-HCV IRES complex

## SUPPLEMENTARY DATA

- SUPPLEMENTARY DATA
