# Supplementary material for: LOOP IIId of the HCV IRES is essential for the structural rearrangement of the 40S-HCV IRES complex
Source: Nucleic Acids Res. 2015 Nov 30;44(3):1309–25. doi: 10.1093/nar/gkv1325 (PMC4756818; doi:10.1093/nar/gkv1325)
Supplement: SUPPLEMENTARY DATA [file supp_gkv1325_nar-02666-y-2015-File010.pdf]

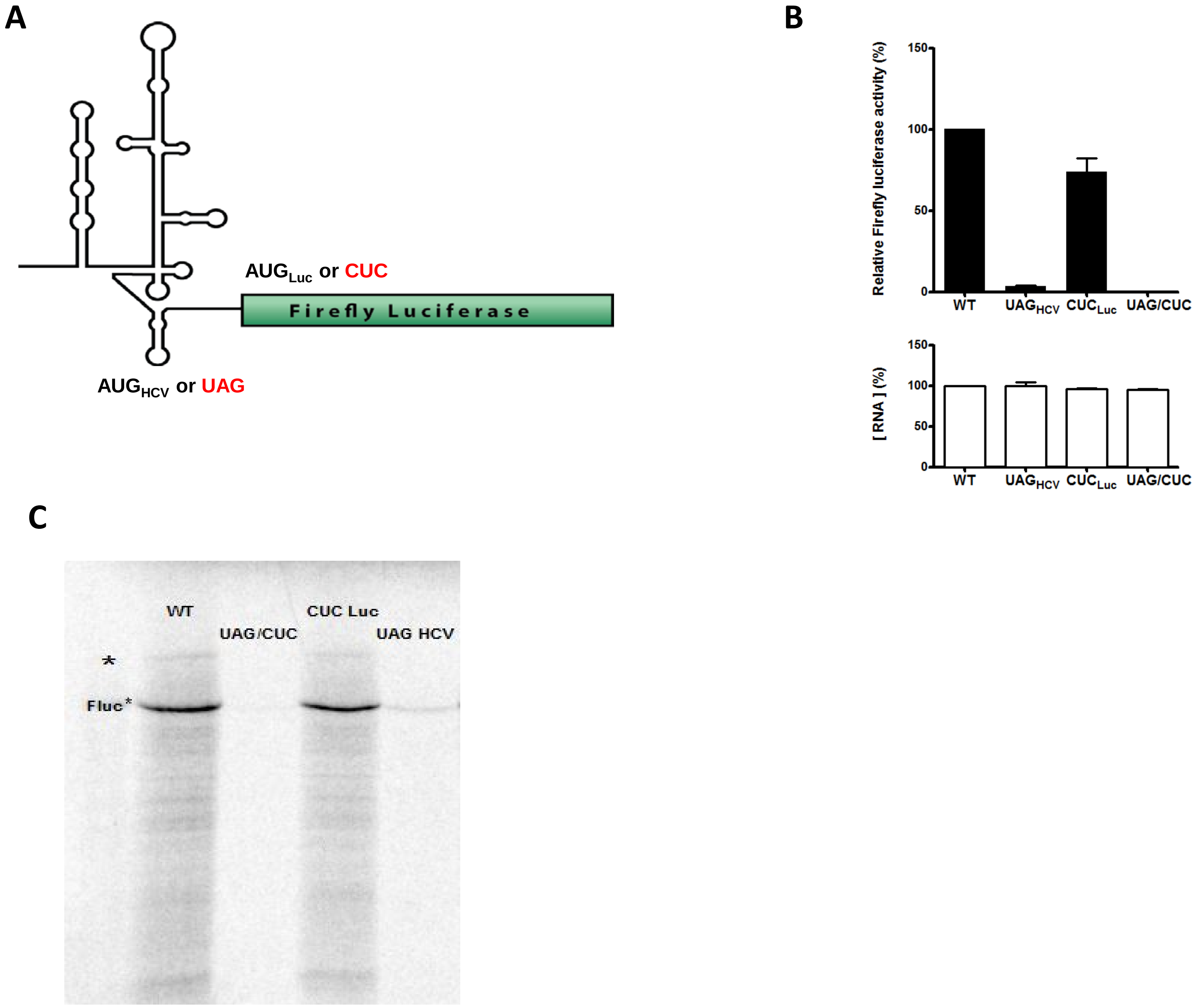

Supplementary Figure S1

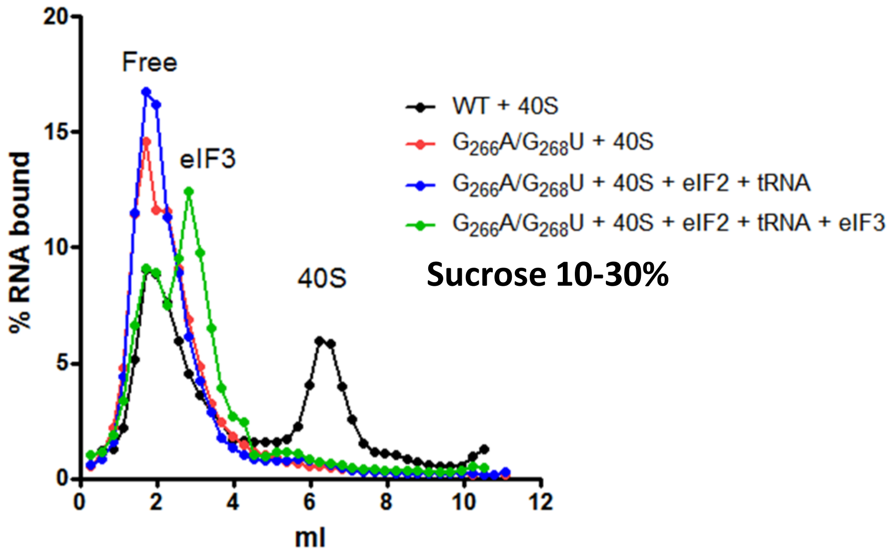

Supplementary Figure S2

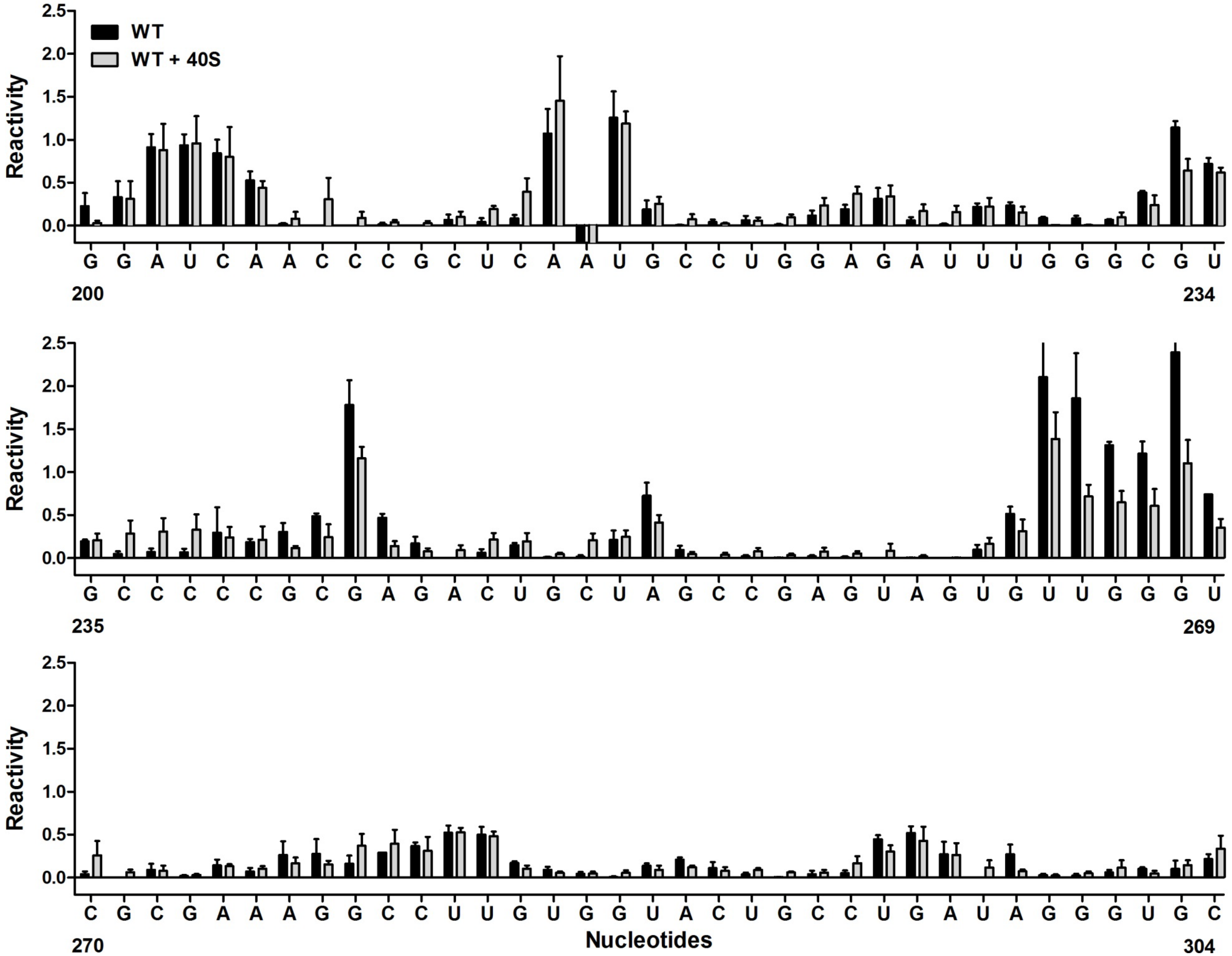

Supplementary Figure S3A

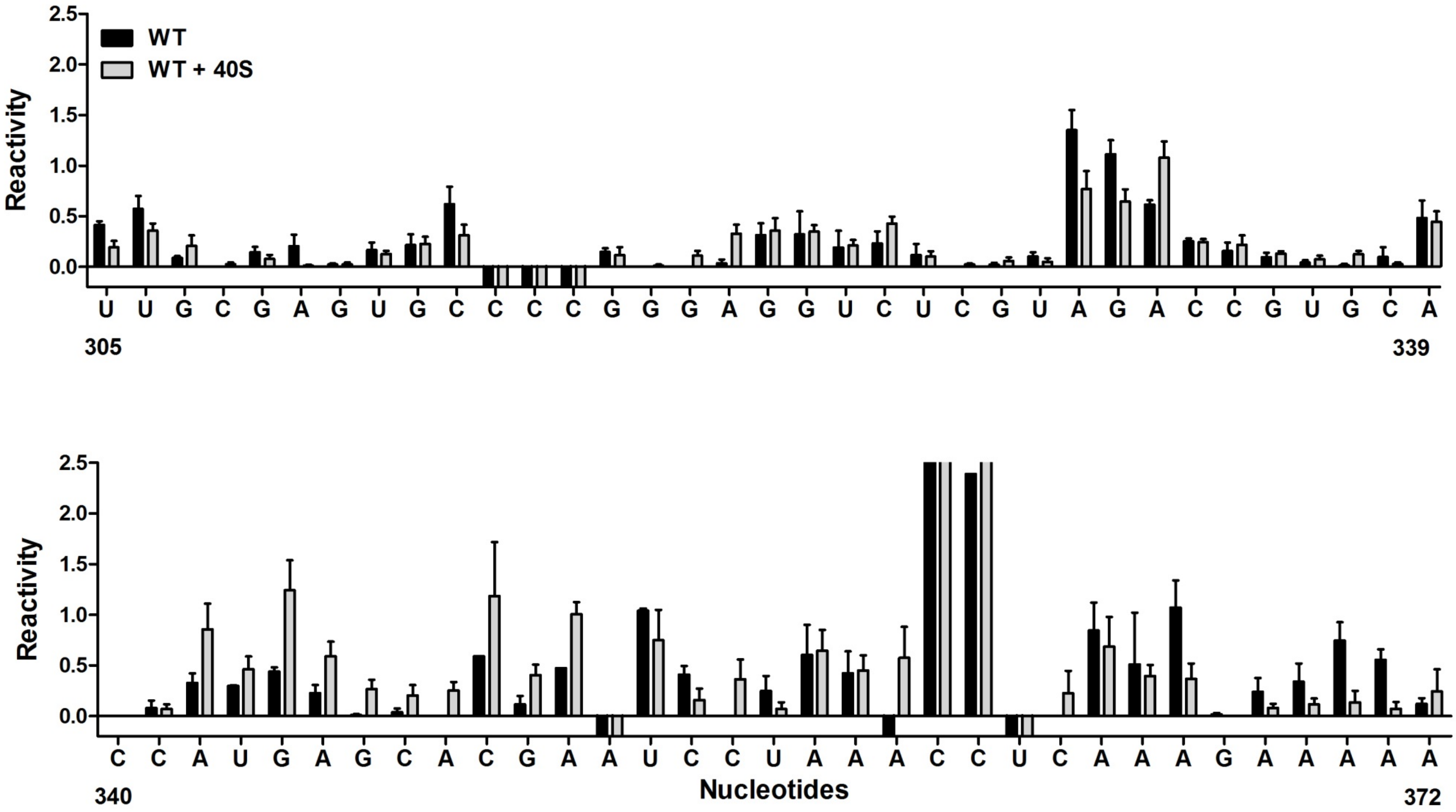

Supplementary Figure S3A

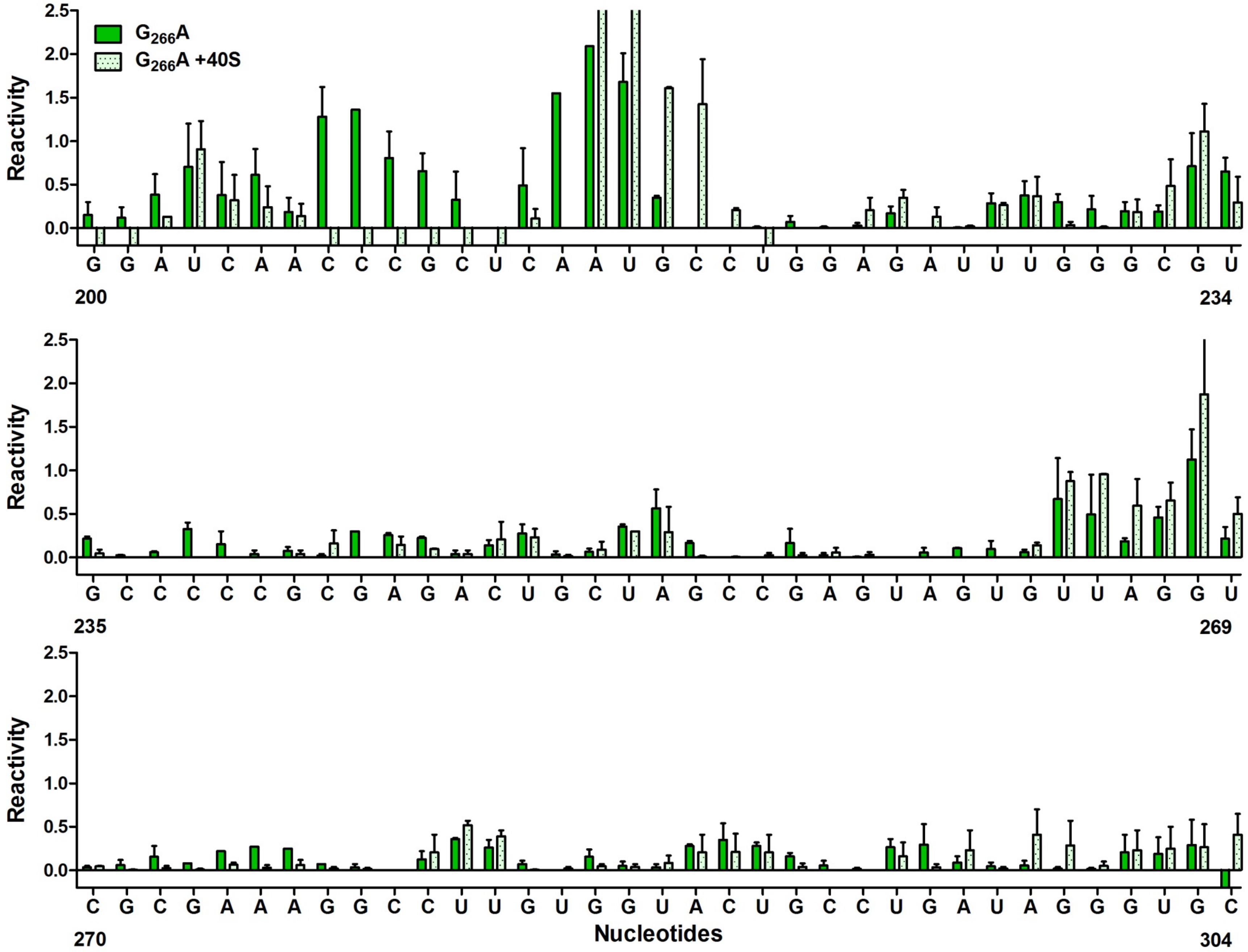

Supplementary Figure S3B

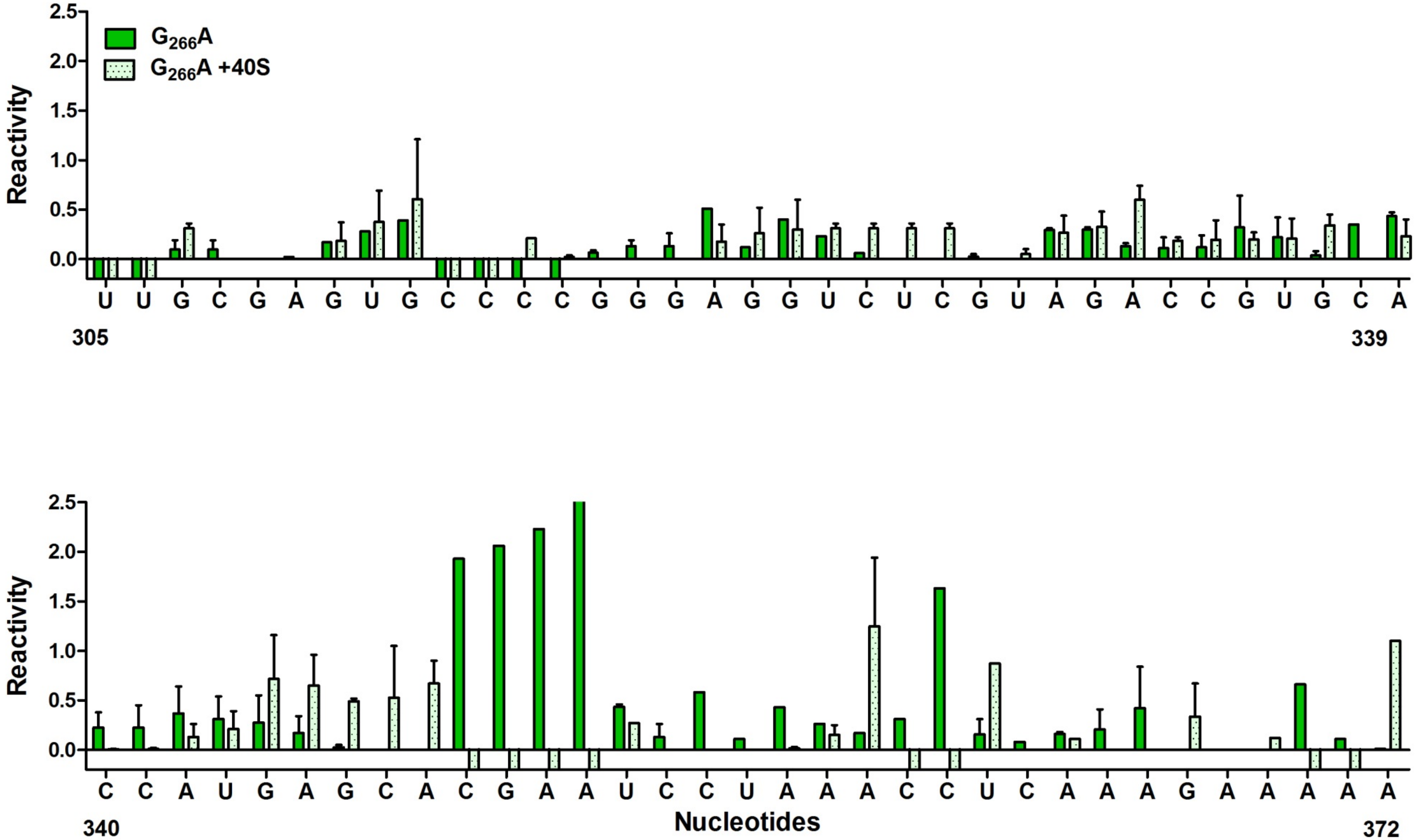

Supplementary Figure S3B

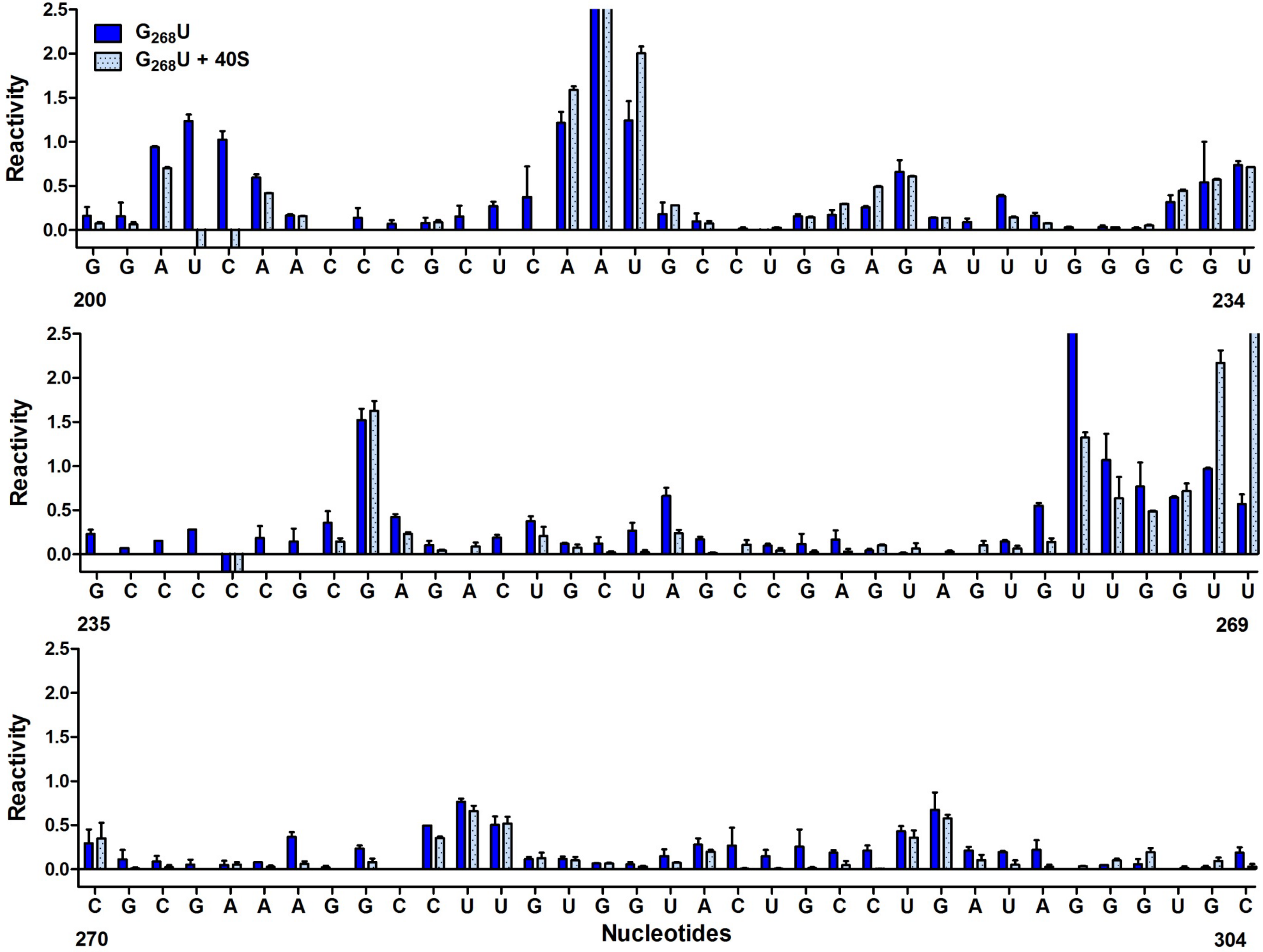

Supplementary Figure S3C

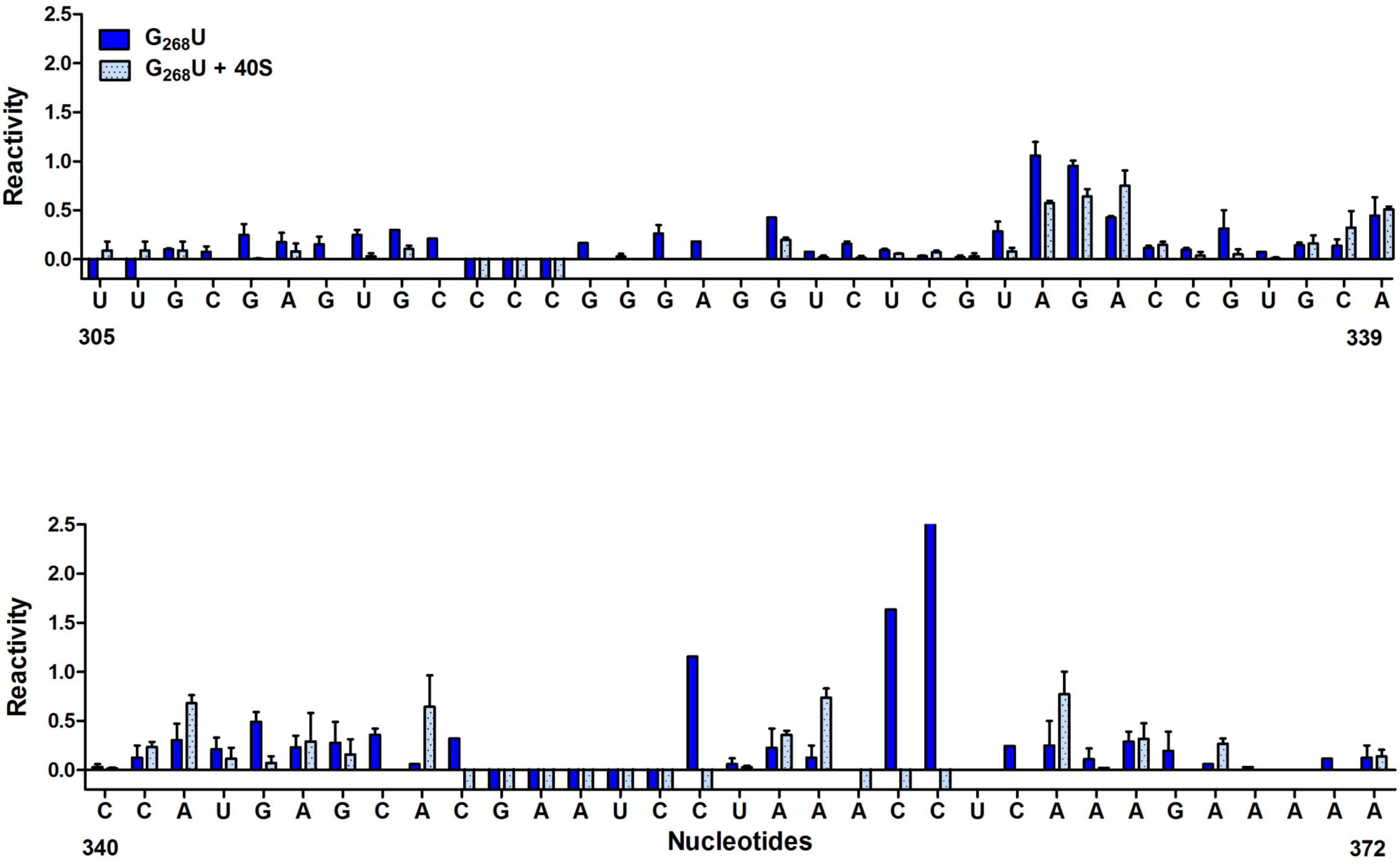

Supplementary Figure S3C

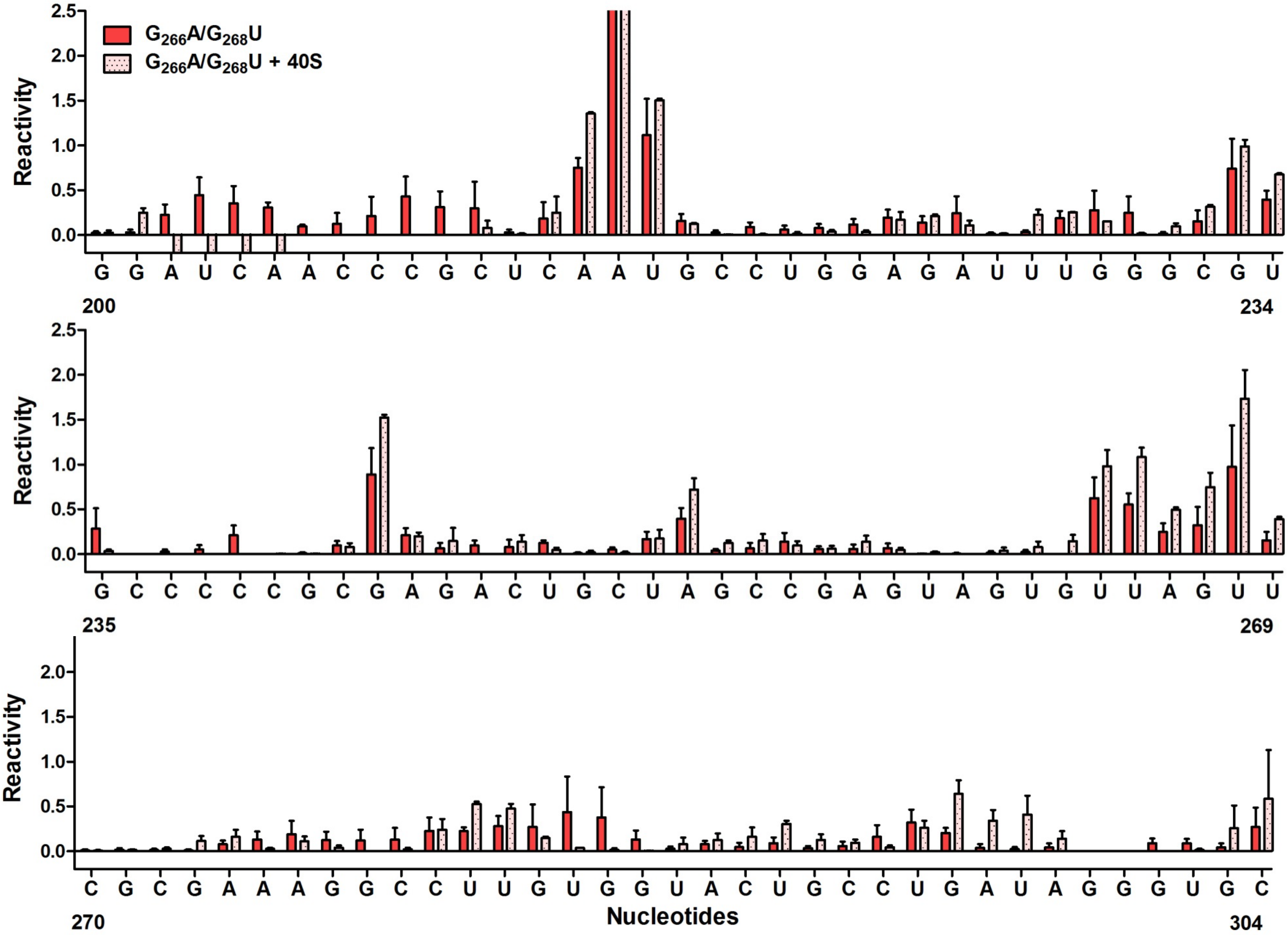

Supplementary Figure S3D

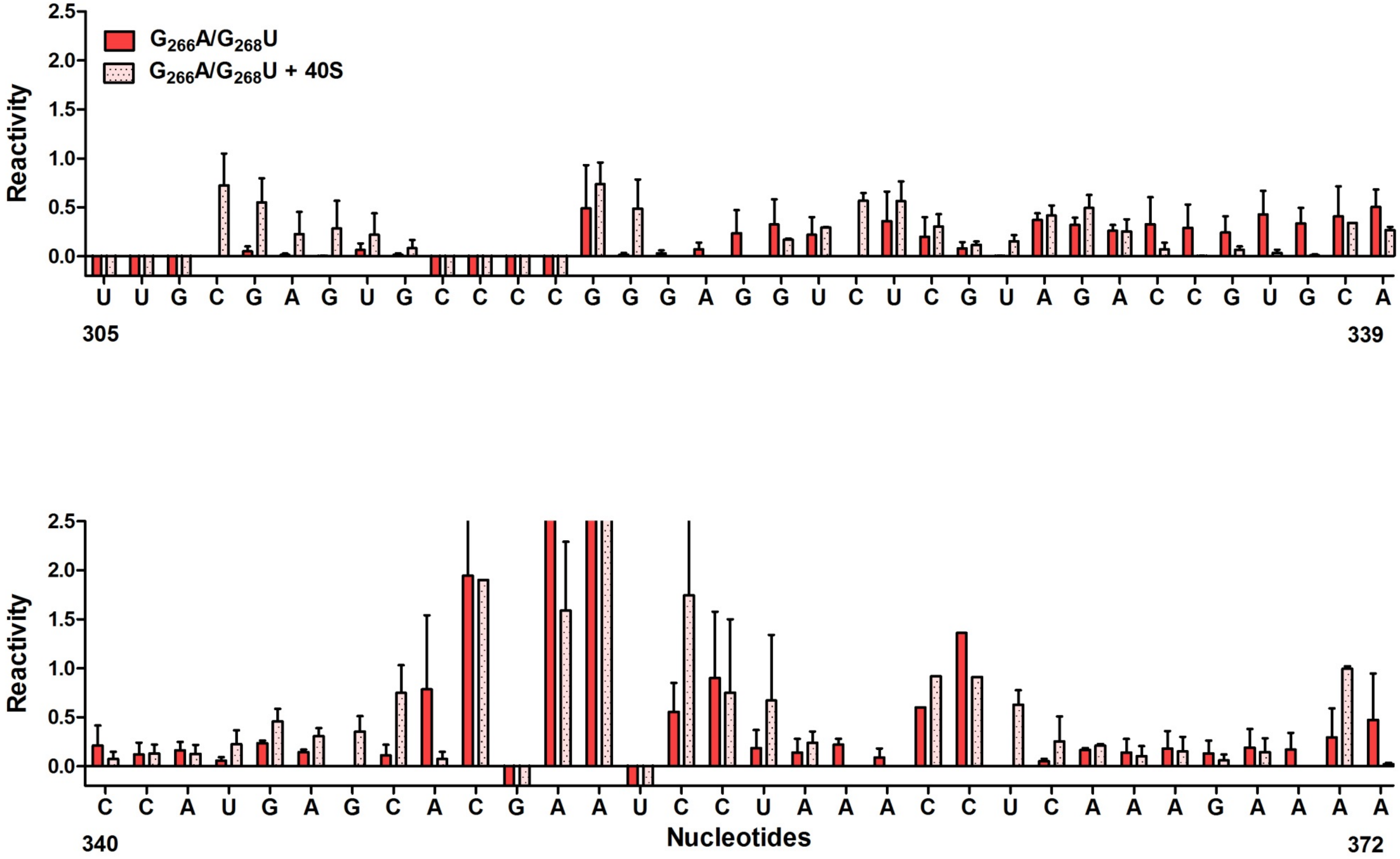

Supplementary Figure S3D

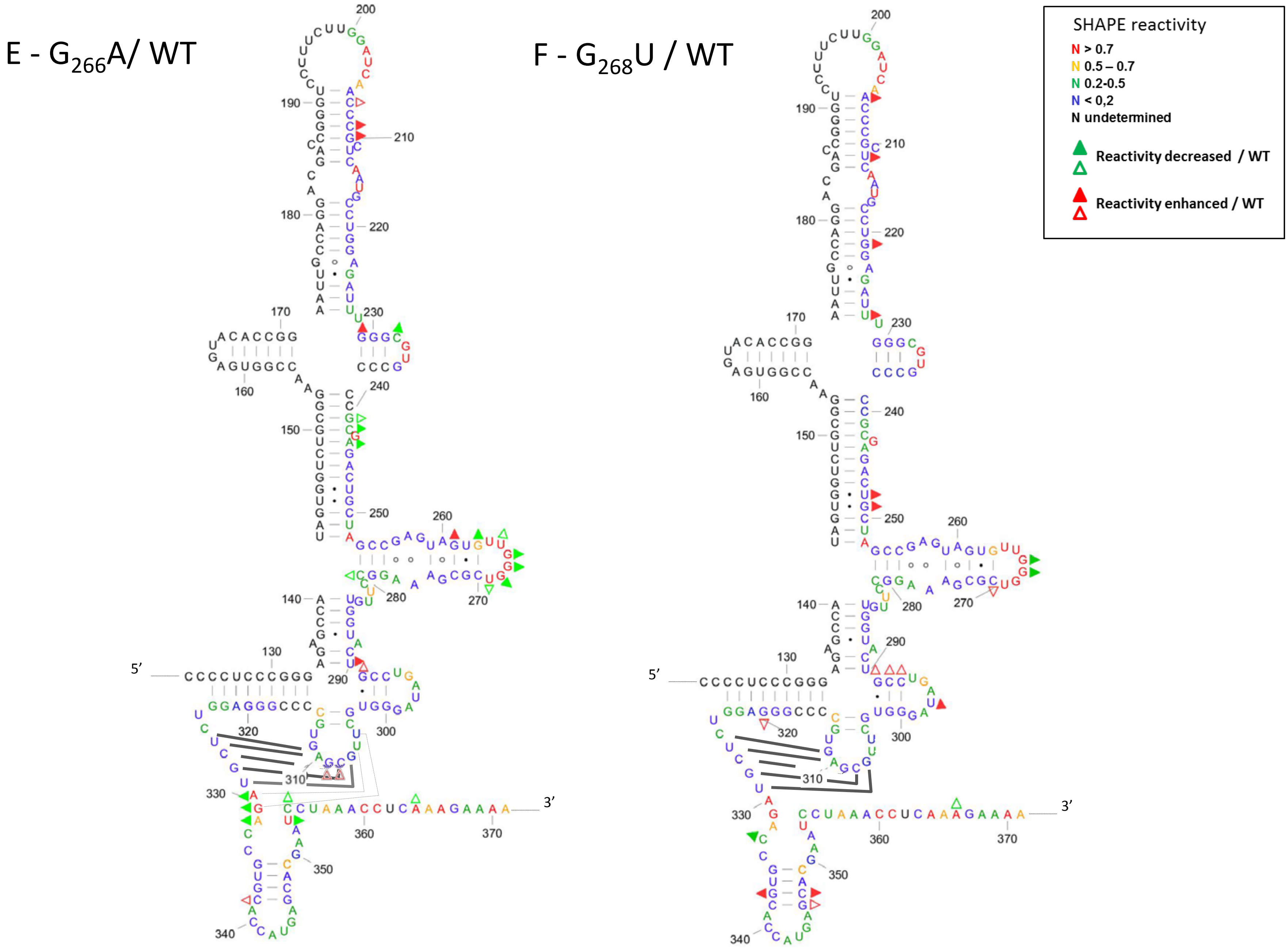

Supplementary Figures S3 E-F

G - DM $G_{266}A$ - $G_{268}U$/WT

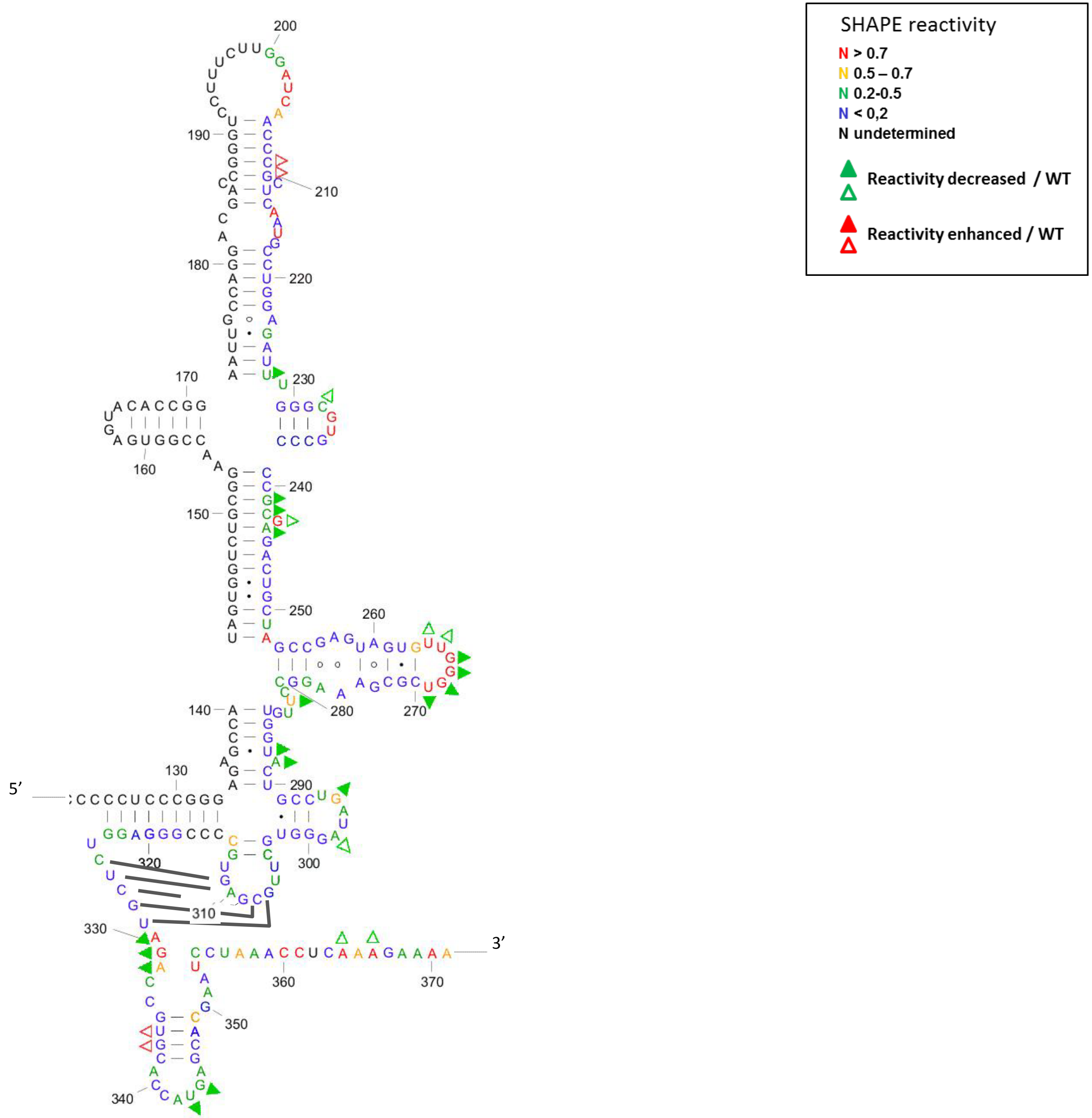

Supplementary Figure S3G

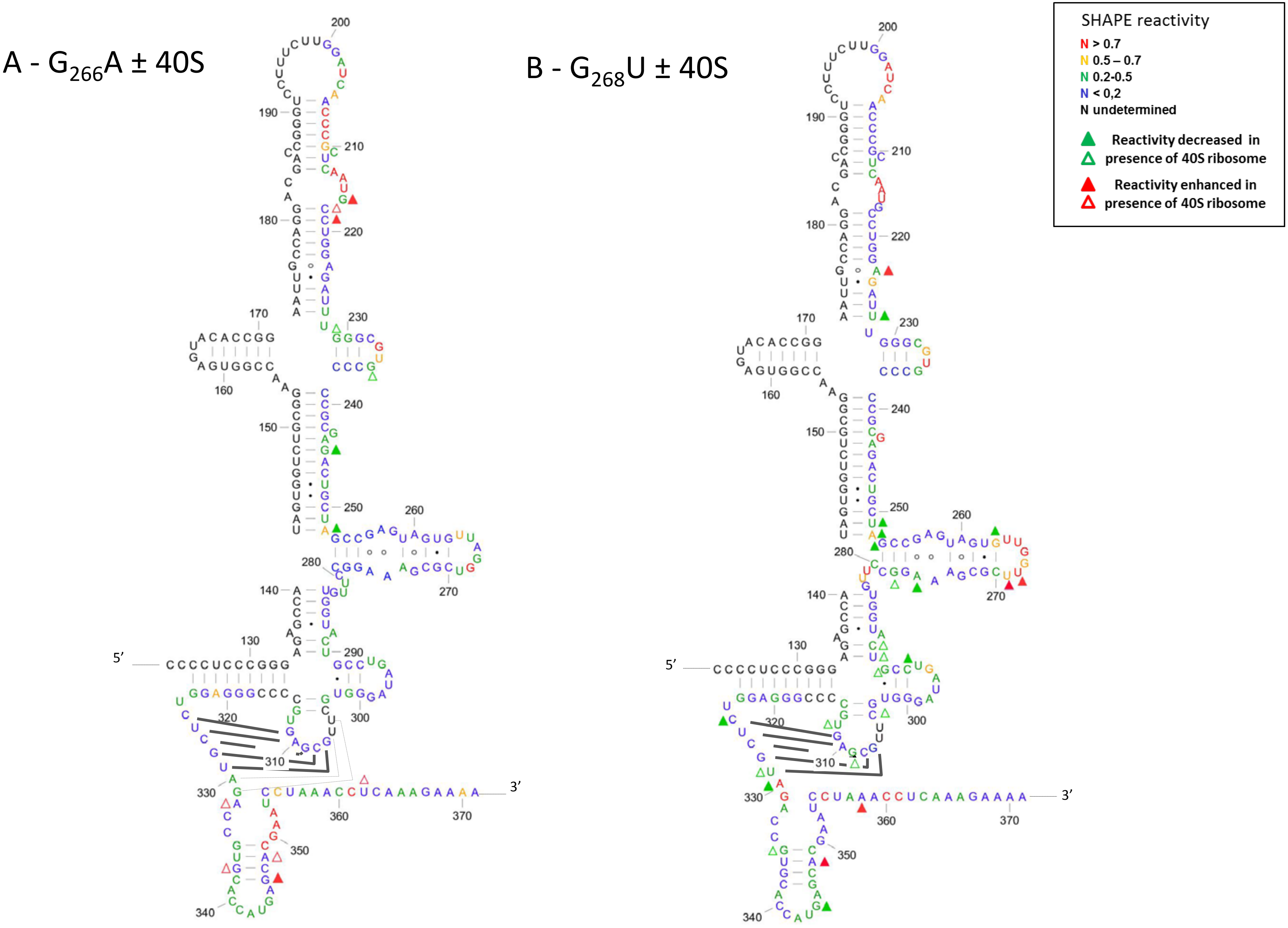

Supplementary Figure S4 A-B

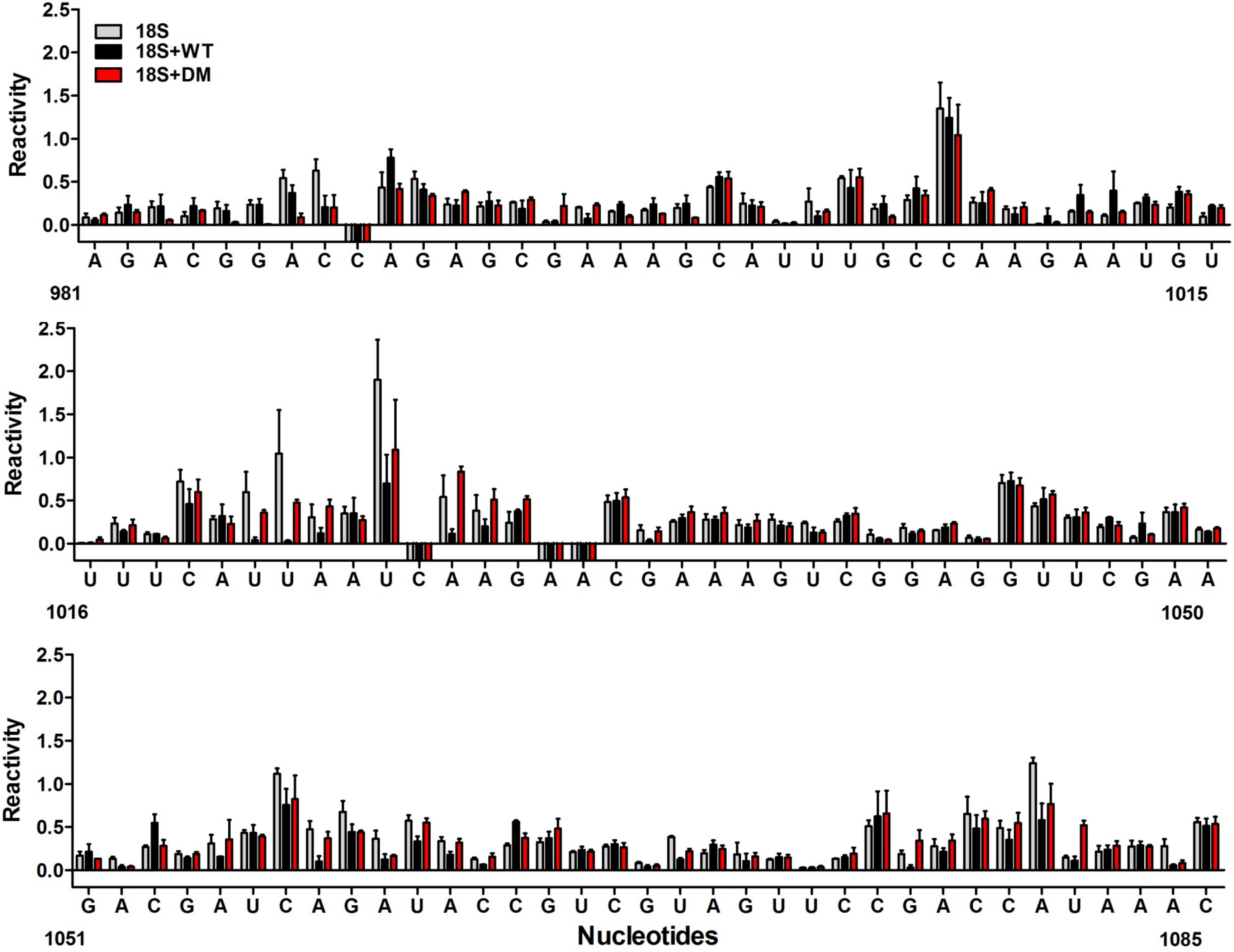

Supplementary Figure S5

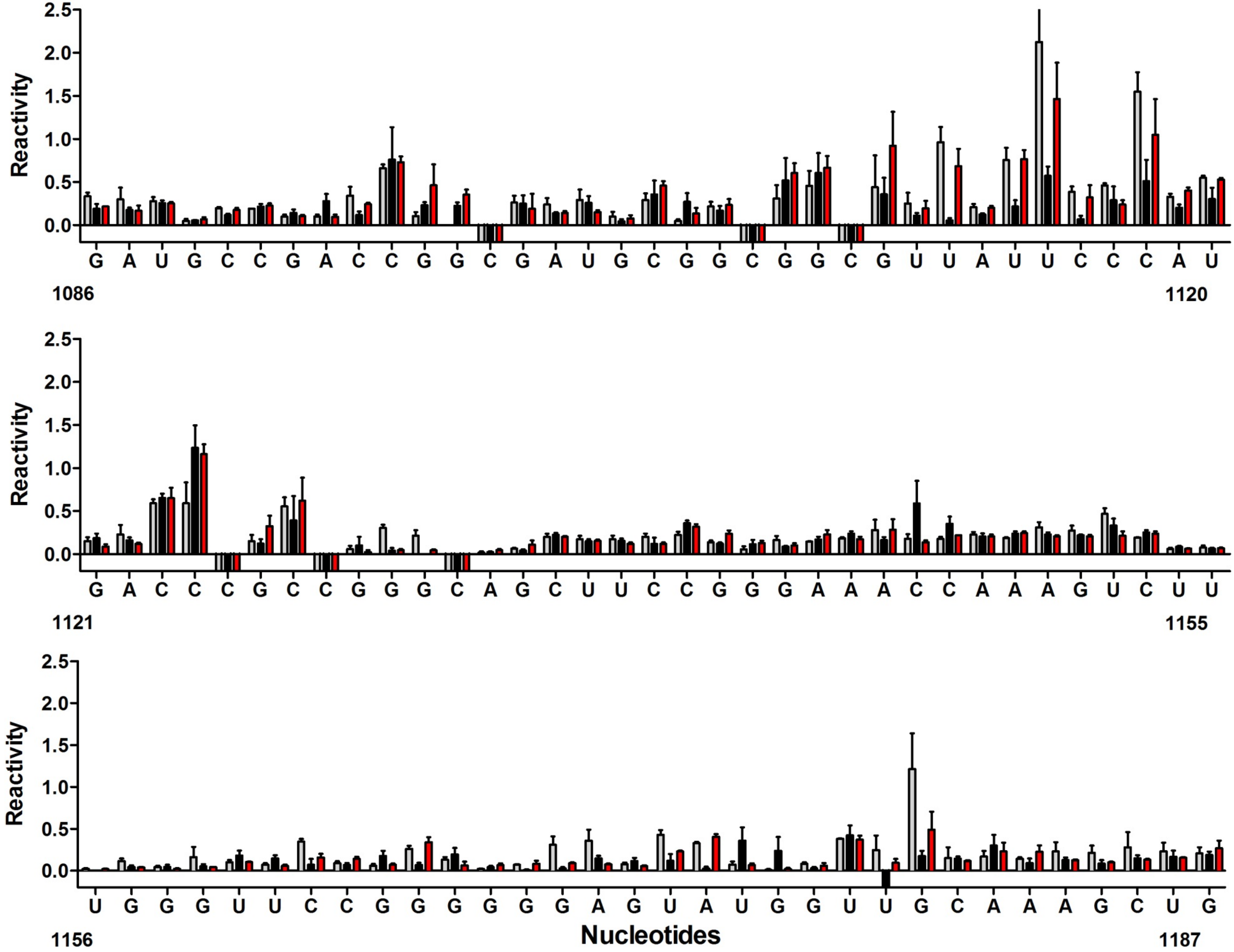

Supplementary Figure S5

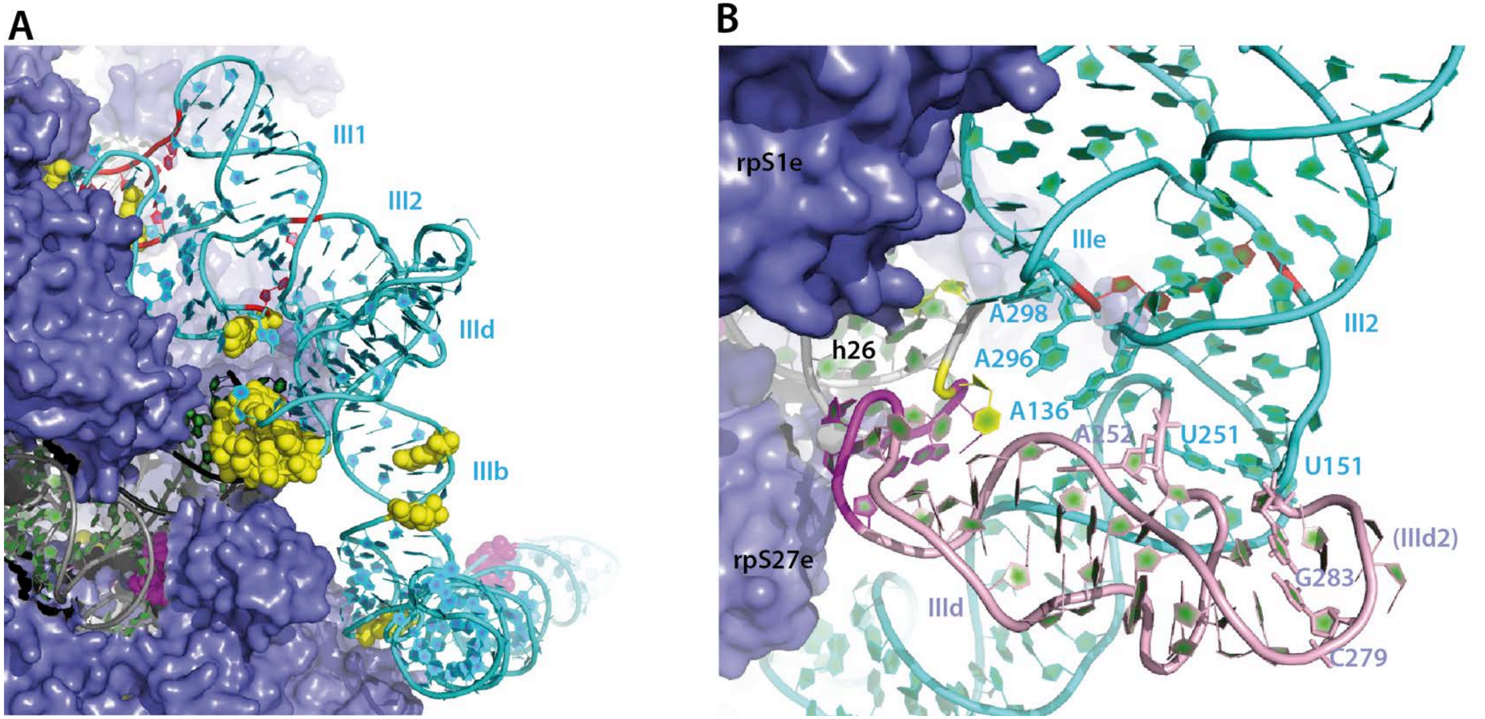

Supplementary Figures S6A - B

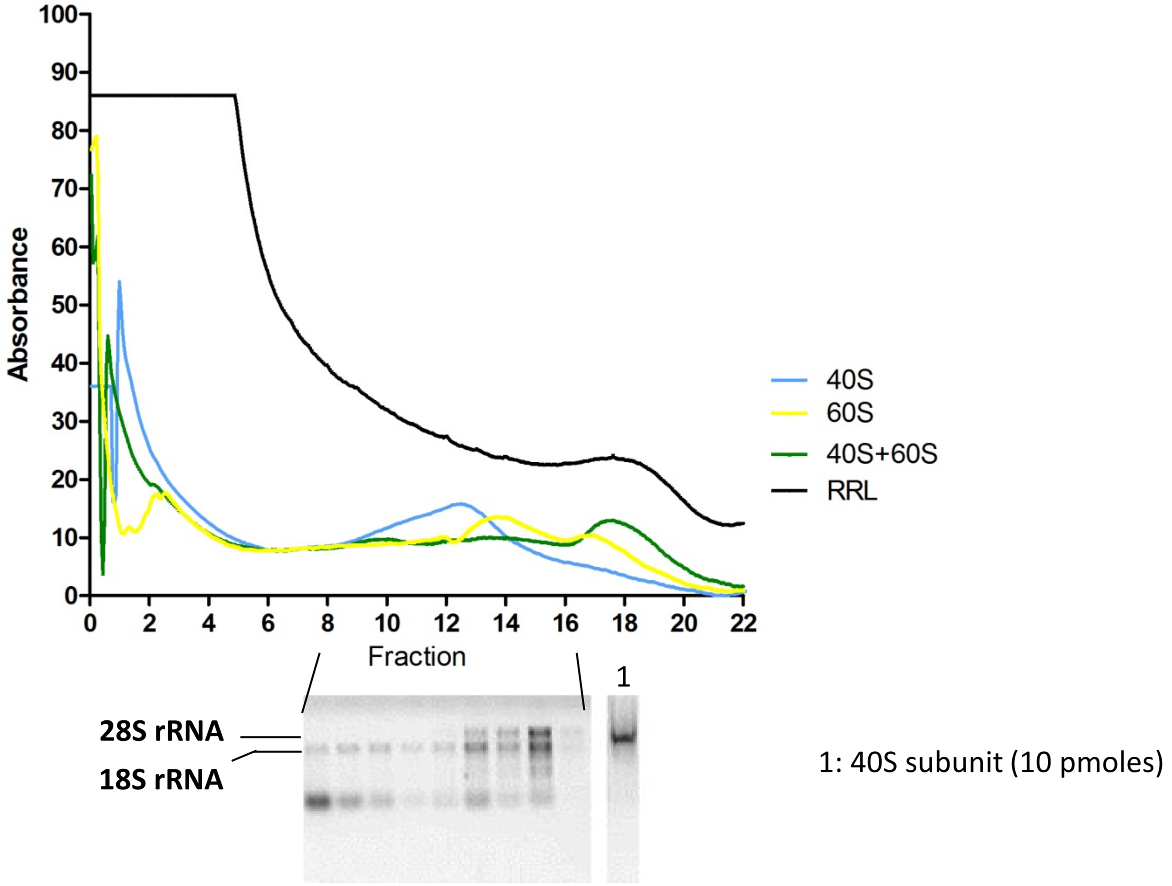

Supplementary Figure S7

Supplementary figures Legends

Supplementary Figure S1: **Replacement of the HCV ORF by the Firefly Luciferase maintains translation initiation at the authentic AUG codon**. (**A**) Schematic representation of the constructs used in this study. The first 48 nucleotides of HCV native open reading frame (green) were conserved to avoid disrupting potential sequences or structural determinants involving nucleotides downstream the initiation codon. (**B**) RNAs (1 pmol) presenting AUG_{HCV} and AUG_{Luc} (WT) or mutated for one initiation codon (UAG_{HCV} and CUC_{Luc}) or for both (UAG/ CUC) were *in vitro* translated as indicated in the method section. Firefly luciferase activity was measured and expressed relative to the WT control (upper panel). The amount of RNA translated was ascertained by qRT-PCR and expressed relative to the WT control. The results are the mean of at least three independent experiments ± standard deviation. **(C)** RNAs (1 pmol) presenting AUG_{HCV} and AUG_{Luc} (WT) or mutated for one initiation codon (UAG_{HCV} and CUC_{Luc}) or for both (UAG/CUC) were translated *in vitro* in RRL in presence of ^{35}S-Methionine as indicated in the method section. Products were resolved on a 12% SDS-PAGE. Translation of the F-Luc*protein is dependent on the integrity of AUG_{HCV} integrity. Of note this holds true for the * protein indicating that this band results from an artefactual migration of the bona fide protein.

Supplementary Figure S2: Fractionation by 10-30% sucrose density gradient of 2 pmoles of ^{32}P labeled WT in the presence of excess purified ribosomal 40S (400 nM) (black) or DM RNA ($G_{266}A/G_{268}U$, colored) in the presence of an excess of purified ribosomal 40S (400 nM) (red), and of purified eIF2 (250 nM); charged $tRNA_{Met}^{i}$ (250 nM) and GTP (1 mM) (blue) and of an excess of purified eIF3 (200 nM) (green).

Supplementary figure S3A: SHAPE probing of WT HCV-IRES in the presence of ribosomal 40S. Histogram bars represent the value of the 1M7 reactivity as calculated with QuSHAPE. The 1M7 reactivity was obtained in the absence (black bars) or presence (grey bars) of ribosomal 40S. Positions for which the reactivity could not be determined because of an unspecific reverse transcriptase stop are indicated with a small negative value (-0.2). Nucleotides are numbered from the native 5' terminus. Reactivity values are the mean value from three independent experiments that were carried out using two different preparations of both 40S ribosomal subunit and IRESes RNA. Error bars are the s.e.m. of three independent experiments.

Supplementary Figure S3B: SHAPE probing of the $G_{266}A$ Loop IIId mutant in the presence of ribosomal 40S. Histogram bars represent the value of the 1M7 reactivity as calculated with QuSHAPE. The 1M7 reactivity was obtained in the absence (green) or presence (dotted green) of ribosomal 40S. Positions for which the reactivity could not be determined because of an unspecific

reverse transcriptase stop are indicated with a small negative value (-0.2). Nucleotides are numbered from the native 5' terminus. Reactivity values are the mean value from three independent experiments that were carried out using two different preparations of both 40S ribosomal subunit and IRESes RNA. Error bars are the s.e.m. of three independent experiments.

Supplementary Figure S3C: SHAPE probing of the G_{268}U Loop IIId mutant in the presence of ribosomal 40S. Histogram bars represent the value of the 1M7 reactivity as calculated with QuSHAPE. The 1M7 reactivity was obtained in the absence (blue) or presence (dotted blue) of ribosomal 40S. Positions for which the reactivity could not be determined because of an unspecific reverse transcriptase stop are indicated with a small negative value (-0.2). Nucleotides are numbered from the native 5' terminus. Reactivity values are the mean value from three independent experiments that were carried out using two different preparations of both 40S ribosomal subunit and IRESes RNA. Error bars are the s.e.m. of three independent experiments.

Supplementary Figure S3D: SHAPE probing of the G_{266}A/G_{268}U Loop IIId double mutant in the presence of ribosomal 40S. Histogram bars represent the value of the 1M7 reactivity as calculated with QuSHAPE. The 1M7 reactivity was obtained in the absence (red) or presence (dotted red) of ribosomal 40S. Positions for which the reactivity could not be determined because of an unspecific reverse transcriptase stop are indicated with a small negative value (-0.2). Nucleotides are numbered from the native 5' terminus. Reactivity values are the mean value from three independent experiments that were carried out using two different preparations of both 40S ribosomal subunit and IRESes RNA. Error bars are the s.e.m. of three independent experiments.

Figure S3 E-G: SHAPE probing of the mutant IRESes compared to the SHAPE probing of the WT IRES Relative reactivity calculated with QuSHAPE for the WT construct are reported on the secondary structure of the IRES. The nucleotides are numbered from the viral transcription initiation start. Only domain III is represented, although the experiment was carried out on the Fluc construct containing domain II and the Fluc coding region (see material and methods). The color code represents the reactivity of each nucleotide in the WT construct as specified in the box. Reactivity values are the mean value from three independent experiments that were carried out using two different preparations of both 40S ribosomal subunit and IRESes RNA. Triangles mark position where the reactivity is significantly altered as compare to the WT IRES. The green triangles represent positions for which the reactivity is decreased in the considered mutant (full triangle: t-test<0.05, ratio r (without 40S/with 40S) >1.5, absolute value of the difference d (|without 40S – with 40S|) >0.1; hollow triangle t<0.15, r>2.25, d>0.15). The red triangles marks positions where the reactivity is enhanced in presence of 40S ribosomal subunit (full triangle t<0.05, r>0.6, d>0.1; hollow triangle

t<0.15, r>0.4, d>0.15). Figures were drawn using XRNA (http://rna.ucsc.edu/rnacenter/xrna/xrna.html). (E) Reactivity modifications observed for the $G_{266}A$ mutant. (F) Reactivity modifications observed for the $G_{268}U$ mutant. (G) Reactivity modifications observed for the $G_{266}A/G_{268}U$ double mutant.

Figure S4: SHAPE reactivity of the $G_{266}A$ **(A) and the** $G_{268}U$ mutant **IRESes (B) in presence and absence of the 40S ribosomal subunit (400 nM).** Relative reactivity calculated with QuSHAPE for the two mutant IRESes are reported on the secondary structure of the $G_{266}A$ and $G_{268}U$ IRES. The nucleotides are numbered from the viral transcription initiation start. Only domain III is represented, although the experiment was carried out on the Fluc construct containing domain II and the Fluc coding region (see material and methods). The color code represents the reactivity of each nucleotide as specified in the box. Reactivity values are the mean value from three independent experiments that were carried out using two different preparations of both 40S ribosomal subunit and IRESes RNA. Triangles represent footprints observed in presence of the ribosome. The green triangles represent positions for which the reactivity is decreased in presence of 40S (full triangle: t-test<0.05, ratio r (without 40S/with 40S) >1.5, absolute value of the difference d (| without 40S – with 40S|) >0.1; hollow triangle t<0.15, r>2.25, d>0.15). The red triangles marks positions where the reactivity is enhanced in presence of 40S ribosomal subunit (full triangle t<0.05, r>0.6, d>0.1; hollow triangle t<0.15, r>0.4, d>0.15). Figures were drawn using XRNA (http://rna.ucsc.edu/rnacenter/xrna/xrna.html).

Supplementary Figure S5: SHAPE probing of the ribosomal 18S. Histogram bars represent the value of the 1M7 reactivity as calculated with QuSHAPE. The 1M7 reactivity was obtained in the absence (grey) or in the presence of the WT IRES (black) or the $G_{266}A/G_{268}U$ Loop IIId double mutant IRES (DM, red). Positions for which the reactivity could not be determined because of an unspecific reverse transcriptase stop are indicated with a small negative value (-0.2). Nucleotides are numbered from the native 5' terminus. Reactivity values are the mean value from three independent experiments that were carried out using two different preparations of both 40S ribosomal subunit and IRESes RNA. Error bars are the s.e.m. of three independent experiments.

Supplementary Figure S6: Detailed views of the HCV IRES model bound to the 40S subunit. (A) Close-up view of a cartoon IRES model oriented as in Figure 7A, showing protections (yellow CPK nucleotides) and enhancements (purple CPK nucleotides) to SHAPE reagents upon binding to the rRNA. The two pseudoknots are displayed in red. (B) Detail of the structure of domain IIId displaying the h26/IIId loop interaction in the vicinity of rpS27e.On the opposite side of IIId, a IIId2 domain

inspired from the CSFV cryo EM structure results from the proposed pairing in trans between C279 and G283. In between the apical loops of IIId lies the loop E motif, already described elsewhere (Klinck et al. (2000) RNA **6**, 1423-1431). The general context in which the four A residues 136, 252, 296 and 298 get in close proximity of h26 is also visible (Figure 7D).

Supplementary Figure S7: Estimation of the free 40S (or 43S) ribosomal subunit in RRL: 100 µl of reticulocyte lysate, or 250 pmoles of purified 40S particles, or 250 pmoles of purified 40S particles or 250 pmoles of purified 40S particles and 250 pmoles of purified 60S particles pre-assembled in 10 mM $mgCl_2$ as described by lancaster et al. (2006) were run on a 15-50% sucrose gradient as described in Material and methods. OD_{260nm} was monitored during the gradient collection. 200 µl (out of 300µl) of fractions 8 to 16 (corresponding to the 40S peak) of the gradient on which was run the RRL were diluted with an equal volume of NaOAC 0,6 M, SDS 0.2% and 5 mM EDTA and extracted with phenol. RNA were then precipitated by addition of 2,5 volume of EtOH for 2 hours at -20°C. RNA were run on a 1% agarose gel, a control containing 10 pmoles of purified 40S particles was run in lane 1 (Purified 40S were not phenol extracted, this is why the 18S rRNA runs slightly less than in the fractions). 18S rRNA present in each fraction was quantitated using a Fuji –LAS 4000 imaging system taking lane 1 as reference.
